# RNF128 regulates neutrophil infiltration and myeloperoxidase functions to prevent acute lung injury

**DOI:** 10.1038/s41419-023-05890-1

**Published:** 2023-06-21

**Authors:** Pei-Yao Liu, Chih-Yuan Chen, Yu-Lung Lin, Chien-Ming Lin, Wen-Chiuan Tsai, Yu-Ling Tsai, Gu-Jiun Lin, Yu-Guang Chen, Shih-Yun Wang, Rui-Nong Sun, Yu-Chuan Huang, Hung Chang, Ying-Chuan Chen

**Affiliations:** 1 Department of Physiology & Biophysics, National Defense Medical Center, Taipei 114, Taiwan, Republic of China; 2 Department of Thoracic surgery, Tri-Service General Hospital Taipei, National Defense Medical Center, Taiwan, Republic of China; 3 The Ph.D. Program for Translational Medicine, College for Medical Science and Technology, Taipei Medical University, Taipei, 110 Taiwan; 4 International Ph.D. Program for Translational Science, College of Medical Science and Technology, Taipei Medical University, Taipei, 110 Taiwan; 5 Department of Pediatrics, Tri-Service General Hospital, National Defense Medical Center, Taipei, Taiwan, Republic of China; 6 Department of Pathology, Tri-Service General Hospital, National Defense Medical Center, Taipei 114, Taiwan, Republic of China; 7 Graduate Institute of Pathology and Parasitology, National Defense Medical Center, Taipei 114, Taiwan, Republic of China; 8 Department of Biology and Anatomy, National Defense Medical Center, Taipei 114, Taiwan, Republic of China; 9 Division of Hematology/Oncology, Department of Internal Medicine, Tri-Service General Hospital, National Defense Medical Center, Taipei 114, Taiwan, Republic of China; 10 Cancer Institute, University College London, London, UK; 11 School of Pharmacy & Institute Pharmacy, National Defense Medical Center, Taipei, Republic of China; 12 Department of Research and Development, National Defense Medical Center, Taipei, Republic of China

**Keywords:** Acute inflammation, Respiratory tract diseases

## Abstract

Acute lung injury (ALI) is characterised by severe pulmonary inflammation, alveolar-capillary barrier disruption, and pulmonary oedema. Therefore, establishing effective therapeutic targets for ALI prevention is crucial. The present study reports a novel function of RNF128 in regulating LPS-induced ALI. Severe lung damage and increased immune cell infiltration were detected in RNF128-deficient mice. In vitro experiments revealed that RNF128 inhibits neutrophil activation by binding to myeloperoxidase (MPO) and reducing its levels and activity. Moreover, RNF128 regulates alveolar macrophage activation and neutrophil infiltration by interacting with TLR4, targeting it for degradation, and inhibiting NF-κB activation, hence decreasing pro-inflammatory cytokines. Our results demonstrate for the first time that RNF128 is a negative regulator of MPO and TLR4 in neutrophils and alveolar macrophages, respectively. However, AAV9-mediated RNF128 overexpression alleviated lung tissue damage and reduced inflammatory cell infiltration. Thus, RNF128 is a promising therapeutic candidate for pharmacological interventions in ALI.

## Introduction

Acute lung injury (ALI) and acute respiratory distress syndrome (ARDS) are life-threatening respiratory system diseases characterised by severe pulmonary inflammation, impaired alveolar-capillary barrier, and pulmonary oedema, resulting in high mortality [1, 2]. The molecular mechanisms of ALI progression have not been fully explored; however, alveolar macrophages and neutrophils play crucial roles in the pathogenesis of ALI [3, 4]. Activated neutrophils and alveolar macrophages release excessive pro-inflammatory cytokines such as tumour necrosis factor-α (TNF-α), interleukin 6 (IL-6), and interleukin-1β (IL-1β), and trigger a local inflammatory response, damaging the lung epithelium and endothelial cells [5]. Myeloperoxidase (MPO) is a haem-containing azurophilic granule enzyme mostly expressed in neutrophils [6]. Activated neutrophils release MPO, which enhances the inflammatory response and tissue damage during ALI [7]. Lipopolysaccharide (LPS), a major constituent of the outer membrane of gram-negative bacteria, promotes ALI [8, 9]. After LPS stimulation, immune cells activate TLR4-dependent signalling and release many cytokines. TLR4 can activate transcription factor nuclear factor-κB (NF-κB) by inhibiting IκB proteins and promoting pro-inflammatory cytokine expression [10, 11]. Although studies have implicated TLR4 signalling in the progression of ALI [12, 13], the underlying molecular mechanisms remain to be elucidated.

RNF128, RING finger protein 128, also known as Grail (a gene related to anergy in lymphocytes), is a type I transmembrane E3 ligase that regulates CD4+ cell tolerance and Treg function [14, 15]. Most studies of RNF128 have focused on the differentiation, proliferation and allergic responses of T lymphocytes [16–18]. Recently, RNF128 was identified as a mediator of sirtuin 1 (sirt1) and shown to regulate the progression of hepatic steatosis [19]. RNF128 mediates adipocyte differentiation and diet-induced obesity by regulating PPARγ levels [20]. Moreover, RNF128 regulates metabolism, cell cycle and apoptosis [17, 20–22].

Recently, we reported that RNF128-deficient mice are highly susceptible to LPS-induced sepsis. RNF128 deletion potentiates macrophage activation and organ injury during sepsis [23]. Sepsis is the most common cause of ALI [24]. However, the function of RNF128 in neutrophils and most myeloid cells during the progression of ALI has not been elucidated. This study aimed to investigate the role of RNF128 in ALI pathophysiology using RNF128 knockout (KO) and adeno-associated virus serotype 9 (AAV9)-mediated RNF128-overexpressing mice. The underlying molecular mechanisms by which RNF128 regulates LPS-induced ALI were evaluated. These findings identified a novel function of RNF128 in regulating ALI, suggesting that RNF128 may be an effective therapeutic target for ALI treatment.

## Results

### RNF128 protects against lung damage in the ALI mouse model

ALI comprises two main phases. First, in the initial three days following the onset of ALI/ARDS, the intrapulmonary inflammatory phase reaches its apex. Patients who survive in the acute inflammatory phase advance to the chronic fibroproliferation phase [25]. We investigated the involvement of RNF128 in the biology of ALI/ARDS, specifically its function during the acute inflammatory phase. We evaluated the effect of RNF128 in mouse disease models characterised by an increased neutrophilic inflammatory response and intrapulmonary cytokines.

**Fig. 1:**
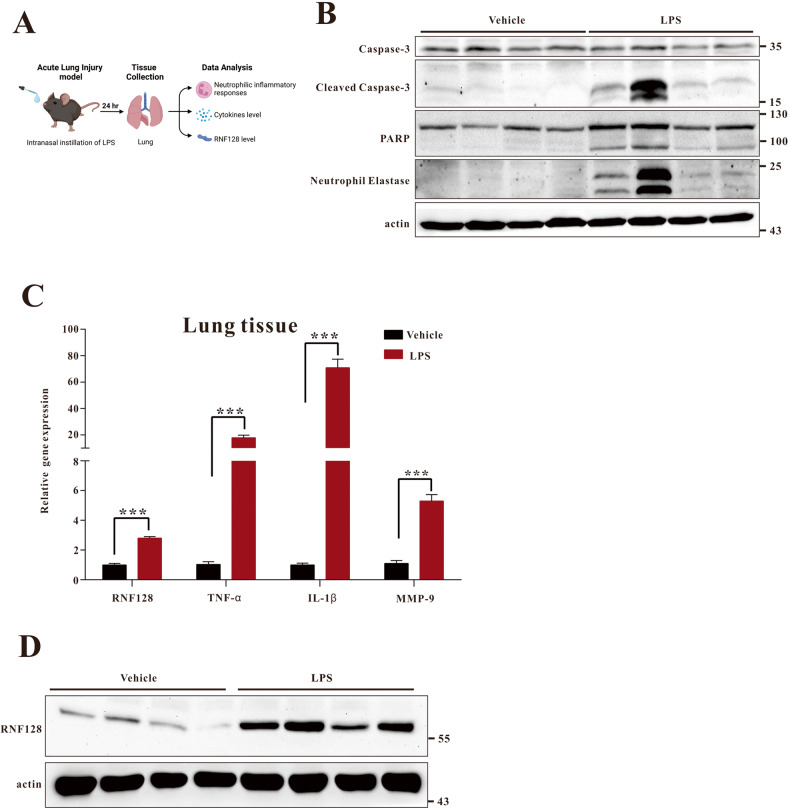
The expression of RNF128 is upregulated in the lungs of LPS-treated mice. **A** Experimental scheme for determining neutrophilic inflammatory response and RNF128 in the ALI model. **B** The expression of Neutrophil Elastase, Caspase 3, Cleaved Caspase 3 and PARP protein in the lungs of mice treated with PBS (WT + PBS, *n* = 4) or LPS (WT + LPS, *n* = 4) for 24 h. **C** The levels of RNF128, TNF-α, IL-1ß and MMP9 mRNAs were measured using Q-PCR. **D** RNF128 protein expression in the lungs of mice treated with PBS (WT + PBS, *n* = 4) or LPS (WT + LPS, *n* = 4) for 24 h. The statistical significance was evaluated using Student’s *t*-test. ^***^P < 0.001, Student’s *t-*test.

**Fig. 2:**
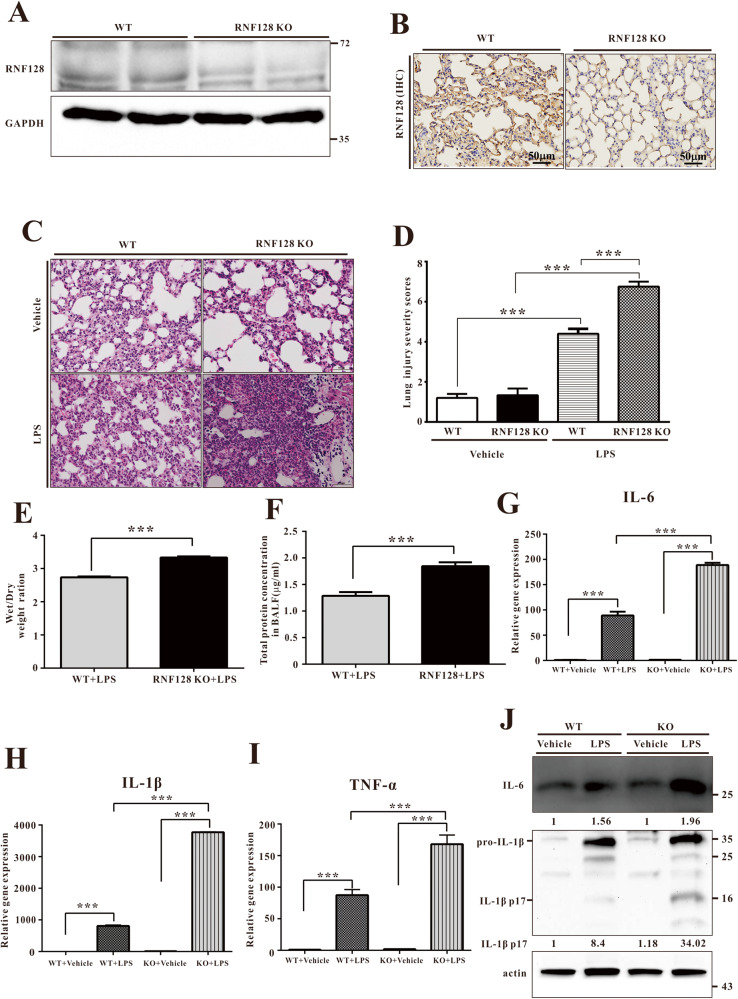
RNF128 deficiency exacerbates LPS-induced ALI. **A** RNF128 protein expression in the lung of mice. **B** Histological images of lung sections stained using RNF128 antibody in the indicated groups. Scale bar, 50 μm. **C**, **D** Mice were administered with saline (control, i.t) or LPS (i.t.) for 24 h. Histological images of lung tissues stained with haematoxylin and eosin (HE) in the indicated groups. Scale bars: 50 μm. An experienced pathologist assigned severity scores to lung injuries. The lung W/D ratio (**E**) and protein level (**F**) in BALF of LPS-treated WT and Grail KO mice. **G**–**I** The expressions of IL-6, IL-1β and TNF-α mRNAs in the lungs were detected using Q-PCR. **J** The levels of IL-6 and IL-1β proteins in the lungs were detected using immunoblotting. Relative immunoblotting signals were inducted. The results shown are representative of at least three independent experiments. The statistical significance was evaluated using one-way ANOVA with Newman–Keuls post hoc test or Student’s *t*-test. *P < 0.05; **P < 0.01; ***P < 0.001.

First, we established a model of intranasal LPS-induced ALI (Fig. 1A). The timing and dose of LPS were selected to simulate the initiation of the acute phase of lung injury. We evaluated the expression of neutrophil elastase, cleaved caspase 3 and cleaved PARP to confirm the lung damage induced by LPS intranasal instillation (Fig. 1B). We also tested the expression of inflammatory cytokines and related genes. All these genes, including TNF-α, IL-1ß and MMP9, were induced in this disease model (Fig. 1C). These results demonstrated the ALI model with LPS intranasal instillation displayed an acute inflammatory response and lung damage. Next, we analysed RNF128 expression in the ALI model. The results showed that RNF128 expression increased in mice with ALI (Fig. 1D and Fig. S1). Based on these results, we hypothesised that RNF128 plays a critical role in the development of ALI. We established RNF128 KO mice and confirmed its expression (Fig. 2A, B). We compared the damage index in WT and RNF128 KO mice after intranasal LPS instillation to confirm our inference. We compared lung tissue histopathology from WT and RNF128 KO mice after ALI induction. We defined a lung injury score based on growth and distribution of damage (alveolar septal thickness, peribronchial and perivascular cuffing, congestive capillaries and polymorphonuclear leucocyte (PMN) infiltration in the lung tissues) to quantify lung damage. The findings revealed that RNF128-deficient mice displayed more severe lung damage (Fig. 2C, D). We measured oedema and protein leakage in bronchoalveolar lavage fluid (BALF) to estimate pulmonary permeability. We evaluated the accumulation of oedema in WT and RNF128 KO mice with LPS-induced ALI using the wet/dry weight ratio. The higher Wet/Dry ratio suggested pulmonary oedema. The results demonstrated that RNF128 KO mice displayed higher pulmonary oedema than WT mice after ALI induction (Fig. 2E, F). Our findings indicated that RNF128 deficiency enhanced lung damage in the ALI model.

### RNF128 ameliorates inflammatory response in the ALI mouse model

The acute inflammatory phase occurs at the initial stages of the onset of ALI/ARDS in humans [26]. Consequently, efficient management of the intrapulmonary inflammatory response either slows the development of ALI/ARDS or promotes survival [27]. We analysed gene and protein expression in lung tissues from WT and RNF128 KO mice with or without ALI induction. In WT-ALI mice, the expression of inflammatory cytokines, including TNF-α, IL-1ß and IL-6, were considerably upregulated compared to controls. After ALI induction, the inflammatory cytokine levels in RNF128 KO mice were higher than in WT mice (Fig. 2G–J). Neutrophils and other immune cells are recruited to induce lung damage after cytokine production and release into the alveoli. We measured cytokine levels in BALF using ELISA to assess the levels of intrapulmonary cytokines. The data showed that BALF derived from RNF128 mice contained higher levels of inflammatory cytokines (Fig. S2A–D). In LPS-induced ALI mice, loss of RNF128 enhances the levels of pro-inflammatory factors.

### RNF128 targets MPO to mitigate ALI-induced lung damage

**Fig. 3:**
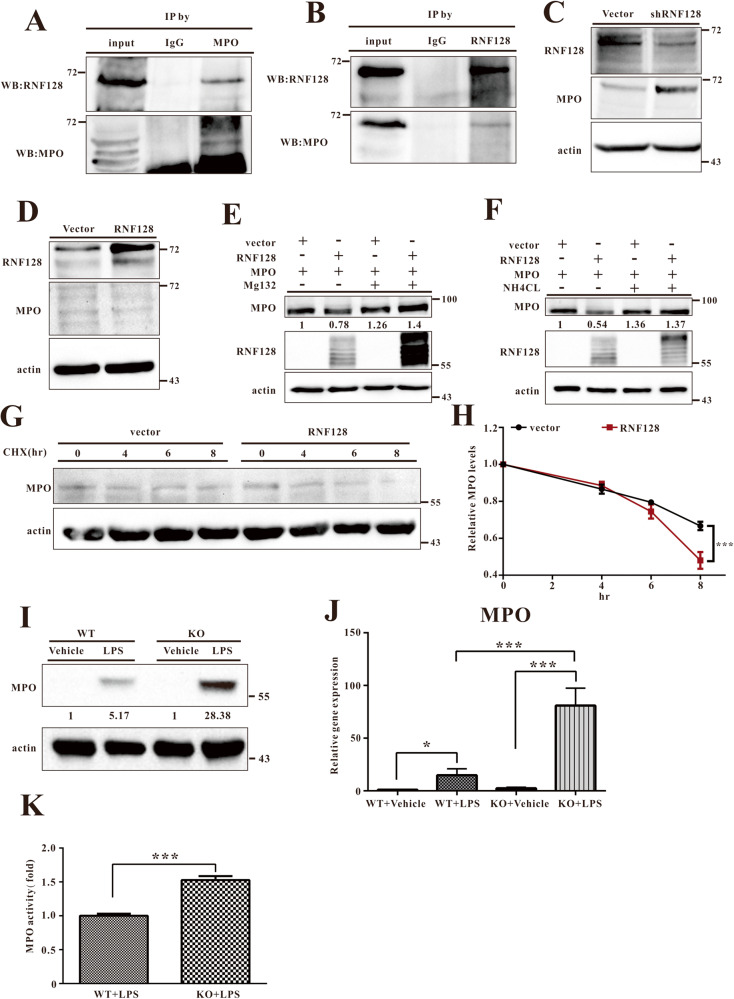
RNF128 interacts with MPO and reduces its level and activity. **A**, **B** Interaction between endogenous RNF128 and MPO. Extracts from differentiated HL-60 cells were immunoprecipitated using anti-RNF128, anti-MPO, or rabbit anti-immunoglobulin G (anti-IgG) antibodies and analysed using anti-RNF128 and anti-MPO antibodies. **C**, **D** Endogenous MPO levels in differentiated HL-60 (RNF128 silencing or overexpression) stable cell lines were detected using immunoblotting. **E** HEK293 cells were transfected with 0.5 μg of vector, RNF128, or MPO and then treated with Mg132 (50 μM) for 8 h. The cells were subsequently harvested and subjected to immunoblotting and the data was quantified using Image J software. **F** HEK293 cells were transfected with 0.5 μg of vector, RNF128, or MPO and then treated with NH_4_CL (50 mM) for 8 h. The cells were subsequently harvested and subjected to immunoblotting and the data was quantified using Image J software. **G**, **H** Differentiated HL-60/vector and differentiated HL-60/RNF128 cells were treated with cycloheximide (CHX, 100 μM) and harvested at the indicated times. Cell lysates were subjected to immunoblotting using the indicated antibodies. **I**, **J** MPO protein levels and mRNA were determined in the lung tissues derived from the indicated groups. Relative immunoblotting signals were inducted. **K** MPO activity was determined in the lung tissues from the indicated groups. The data are presented as mean ± SD. The statistical significance was evaluated using one-way ANOVA with Newman–Keuls post hoc test or Student’s *t*-test. *P < 0.05; ***P < 0.001.

We demonstrated that RNF128 reduces ALI-induced lung damage; however, the underlying mechanism remains unclear. Next, we explored the RNF128 interacting proteins and their network. Immunoprecipitation-mass spectrometry revealed novel RNF128 interacting proteins and their network. The immunoprecipitation-mass spectrometry procedure used in this study is depicted schematically (Fig. S3). Whole lung tissue lysates from WT and WT-LPS mice were immunoprecipitated using the RNF128 antibody. Immunoprecipitated proteins were digested with trypsin and identified using liquid chromatography-tandem mass spectroscopy (LC–MS/MS). We identified 48 distinct proteins that were at least 2-fold more abundant in the WT-LPS mice than the WT control, suggesting that they were RNF128-specific (Table 1). Of these 48 proteins, MPO was the most abundant RNF128-interacting protein after ALI induction. We next confirmed the specific interaction of MPO with RNF128 using co-immunoprecipitation, followed by immunoblotting. Neutrophils play a key role in ALI and ARDS [28]. MPO is the most abundantly expressed protein by human neutrophils for generating chlorinating and other oxidants to kill bacterial and living cells [6]. Therefore, HL-60-derived neutrophils were used to confirm the interaction between RNF128 and MPO. Co-immunoprecipitation experiments using anti-RNF128 and anti-MPO indicated that RNF128 physically interacts with MPO (Fig. 3A, B). Previous studies have shown that RNF128 is an E3 ubiquitin ligase that modifies protein stability and function via its enzymatic activity [19–21, 29]. Next, we determined whether RNF128 could regulate neutrophil MPO expression to improve the MPO-induced damage. We evaluated MPO levels in HL-60-derived neutrophils in the presence or absence of RNF128. We detected elevated MPO expression following RNF128 silencing. In contrast, MPO expression was reduced in the RNF128-overexpressing cells (Fig. 3C, D). Furthermore, we used the proteasome degradation inhibitor, Mg132, or the lysosome degradation inhibitor, ammonium chloride (NH_4_Cl), to determine whether RNF128 degrades MPO in proteasomes or lysosomes. The results showed that either Mg132 or NH_4_Cl could rescue the RNF128-induced reduction in MPO protein expression (Fig. 3E, F). Evaluation of MPO levels following treatment with cycloheximide, a protein synthesis inhibitor, revealed that RNF128 decreased endogenous MPO stability (Fig. 3G, H). In summary, we propose that RNF128 regulates MPO expression through direct interactions.

**Table 1:** The RNF128-involved pathway was identified using IP-Mass.

Accession	Description		WT-LPS	WT	Fold Change
P11247	Myeloperoxidase OS = Mus musculus OX = 10090 GN = Mpo PE = 1 SV = 2 − [PERM_MOUSE]	Mpo	2656.150413	102.1480795	26.00294031
Q9JIK5	Nucleolar RNA helicase 2 OS = Mus musculus OX = 10090 GN=Ddx21 PE = 1 SV = 3 − [DDX21_MOUSE]	Ddx21	566.2804627	28.04	20.1954516
P20918	Plasminogen OS = Mus musculus OX = 10090 GN=Plg PE = 1 SV = 3 − [PLMN_MOUSE]	Plg	560.4548281	29.03	19.30605677
Q9ET01	Glycogen phosphorylase, liver form OS = Mus musculus OX = 10090 GN = Pygl PE = 1 SV = 4 − [PYGL_MOUSE]	Pygl	675.6869767	69.15111269	9.771165645
P61979	Heterogeneous nuclear ribonucleoprotein K OS=Mus musculus OX = 10090 GN = Hnrnpk PE = 1 SV = 1 − [HNRPK_MOUSE]	Hnrnpk	513.0475359	59.73333333	8.588965445
Q9WUU8	TNFAIP3-interacting protein 1 OS = Mus musculus OX = 10090 GN=Tnip1 PE = 1 SV = 1 − [TNIP1_MOUSE]	Tnip1	357.349945	45.72789999	7.814702733
Q99PL5	Ribosome-binding protein 1 OS = Mus musculus OX = 10090 GN=Rrbp1 PE = 1 SV = 2 − [RRBP1_MOUSE]	Rrbp1	458.6563932	61.00301438	7.518585727
Q9CZX8	40 S ribosomal protein S19 OS = Mus musculus OX = 10090 GN=Rps19 PE = 1 SV = 3 − [RS19_MOUSE]	Rps19	287.489367	38.87	7.396176151
Q99K48	Non-POU domain-containing octamer-binding protein OS = Mus musculus OX = 10090 GN = Nono PE = 1 SV = 3 − [NONO_MOUSE]	Nono	311.08064	43.94	7.079668638
P10126	Elongation factor 1-alpha 1 OS = Mus musculus OX = 10090 GN=Eef1a1 PE = 1 SV = 3 − [EF1A1_MOUSE]	Eef1a1	473.3300346	97.66	4.846713441
P26041	Moesin OS = Mus musculus OX = 10090 GN = Msn PE = 1 SV = 3 − [MOES_MOUSE]	Msn	1264.85134	275.8477857	4.58532352
P26039	Talin-1 OS=Mus musculus OX = 10090 GN = Tln1 PE = 1 SV = 2 − [TLN1_MOUSE]	Tln1	199.3702293	46.18	4.317241864
O08692	Neutrophilic granule protein OS = Mus musculus OX = 10090 GN=Ngp PE = 1 SV = 1 − [NGP_MOUSE]	Ngp	1339.731731	324.8263491	4.124455219
Q8VDD5	Myosin-9 OS = Mus musculus OX = 10090 GN=Myh9 PE = 1 SV = 4 − [MYH9_MOUSE]	Myh9	763.8241756	195.3123617	3.910782548
P62245	40S ribosomal protein S15a OS = Mus musculus OX = 10090 GN=Rps15a PE = 1 SV = 2 − [RS15A_MOUSE]	Rps15a	171.2987103	45.35625824	3.776738138
Q8K2I3	Dimethylaniline monooxygenase [N-oxide-forming] 2 OS = Mus musculus OX = 10090 GN = Fmo2 PE = 1 SV = 3 − [FMO2_MOUSE]	Fmo2	111.9302569	31.86906198	3.51219176
P20029	Endoplasmic reticulum chaperone BiP OS=Mus musculus OX = 10090 GN=Hspa5 PE = 1 SV = 3 − [BIP_MOUSE]	Hspa5	408.7148581	121.53	3.363077907
Q6PDM2	Serine/arginine-rich splicing factor 1 OS = Mus musculus OX = 10090 GN = Srsf1 PE = 1 SV = 3 − [SRSF1_MOUSE]	Srsf1	368.0803336	113.558529	3.241327065
P62918	60S ribosomal protein L8 OS = Mus musculus OX = 10090 GN=Rpl8 PE = 1 SV = 2 − [RL8_MOUSE]	Rpl8	402.9989846	127.3756428	3.163862224
Q8R081	Heterogeneous nuclear ribonucleoprotein L OS = Mus musculus OX = 10090 GN = Hnrnpl PE = 1 SV = 2 − [HNRPL_MOUSE]	Hnrnpl	514.2045533	164.5514161	3.124886832
P01872	Immunoglobulin heavy constant mu OS = Mus musculus OX = 10090 GN=Ighm PE = 1 SV = 2 − [IGHM_MOUSE]	Ighm	478.7699937	158.91	3.012837415
Q02105	Complement C1q subcomponent subunit C OS = Mus musculus OX = 10090 GN = C1qc PE = 1 SV = 2 − [C1QC_MOUSE]	C1qc	89.02385808	31.2	2.853328785
P62849	40S ribosomal protein S24 OS = Mus musculus OX = 10090 GN=Rps24 PE = 1 SV = 1 − [RS24_MOUSE]	Rps24	455.8471599	162.5019545	2.805179553
P01864	Ig gamma-2A chain C region secreted form OS = Mus musculus OX = 10090 PE = 1 SV = 1 − [GCAB_MOUSE]		468.2990245	167.4	2.797485212
Q8BMF4	Dihydrolipoyllysine-residue acetyltransferase component of pyruvate dehydrogenase complex, mitochondrial OS = Mus musculus OX = 10090 GN = Dlat PE = 1 SV = 2 − [ODP2_MOUSE]	Dlat	509.7281186	186.46	2.73371296
Q9D0T1	NHP2-like protein 1 OS = Mus musculus OX = 10090 GN = Snu13 PE = 1 SV = 4 - [NH2L1_MOUSE]	Snu13	179.43	66.69	2.690508322
O70133	ATP-dependent RNA helicase A OS=Mus musculus OX = 10090 GN=Dhx9 PE = 1 SV = 2 − [DHX9_MOUSE]	Dhx9	292.4390632	111.8318968	2.614987955
P63276	40 S ribosomal protein S17 OS = Mus musculus OX = 10090 GN=Rps17 PE = 1 SV = 2 − [RS17_MOUSE]	Rps17	156.7504083	61.14	2.563794706
P27546	Microtubule-associated protein 4 OS=Mus musculus OX = 10090 GN=Map4 PE = 1 SV = 3 − [MAP4_MOUSE]	Map4	115.58	45.36	2.548059965
Q9JJI8	60 S ribosomal protein L38 OS=Mus musculus OX = 10090 GN=Rpl38 PE = 1 SV = 3 − [RL38_MOUSE]	Rpl38	107.4458462	42.71	2.515707006
P01899	H-2 class I histocompatibility antigen, D-B alpha chain OS=Mus musculus OX = 10090 GN = H2-D1 PE = 1 SV = 2 − [HA11_MOUSE]	H2-D1	185.7113999	74.82	2.482109061
Q8VIJ6	Splicing factor, proline- and glutamine-rich OS=Mus musculus OX = 10090 GN=Sfpq PE = 1 SV = 1 − [SFPQ_MOUSE]	Sfpq	531.8918203	220.4183318	2.413101559
Q8VEK3	Heterogeneous nuclear ribonucleoprotein U OS = Mus musculus OX = 10090 GN = Hnrnpu PE = 1 SV = 1 − [HNRPU_MOUSE]	Hnrnpu	1144.404106	478.7680874	2.390309915
Q9R0P5	Destrin OS = Mus musculus OX = 10090 GN = Dstn PE = 1 SV = 3 − [DEST_MOUSE]	Dstn	94.6	39.66333333	2.385074376
P50404	Pulmonary surfactant-associated protein D OS = Mus musculus OX = 10090 GN = Sftpd PE = 1 SV = 1 − [SFTPD_MOUSE]	Sftpd	249.7905069	109.321253	2.284921734
Q8BTM8	Filamin-A OS =Mus musculus OX = 10090 GN=Flna PE = 1 SV = 5 − [FLNA_MOUSE]	Flna	844.3285904	372.4891434	2.266719998
P62281	40S ribosomal protein S11 OS = Mus musculus OX = 10090 GN=Rps11 PE = 1 SV = 3 − [RS11_MOUSE]	Rps11	323.7022669	143.4275606	2.256904222
P61327	Protein mago nashi homologue OS = Mus musculus OX = 10090 GN=Magoh PE = 2 SV = 1 − [MGN_MOUSE]	Magoh	111.886991	49.57651523	2.256854692
P62852	40S ribosomal protein S25 OS = Mus musculus OX = 10090 GN=Rps25 PE = 1 SV = 1 − [RS25_MOUSE]	Rps25	258.4070323	116.2386753	2.223072756
P48962	ADP/ATP translocase 1 OS = Mus musculus OX = 10090 GN=Slc25a4 PE = 1 SV = 4 − [ADT1_MOUSE]	Slc25a4	198.1835486	89.31673657	2.218884794
P43276	Histone H1.5 OS = Mus musculus OX = 10090 GN=Hist1h1b PE = 1 SV = 2 − [H15_MOUSE]	Hist1h1b	486.4603075	223.1484272	2.179985373
P01867	Ig gamma-2B chain C region OS = Mus musculus OX = 10090 GN=Igh-3 PE = 1 SV = 3 − [IGG2B_MOUSE]	Igh-3	883.5515727	408.4762325	2.163042797
P63017	Heat shock cognate 71 kDa protein OS = Mus musculus OX = 10090 GN =Hspa8 PE = 1 SV = 1 − [HSP7C_MOUSE]	Hspa8	639.4902806	295.9818624	2.160572528
Q6ZWV3	60S ribosomal protein L10 OS = Mus musculus OX = 10090 GN=Rpl10 PE = 1 SV = 3 − [RL10_MOUSE]	Rpl10	604.5828163	285.6226998	2.116718373
P01837	Immunoglobulin kappa constant OS = Mus musculus OX = 10090 GN = Igkc PE = 1 SV = 2 − [IGKC_MOUSE]	Igkc	611.1369241	295.1554889	2.070559237
P51410	60S ribosomal protein L9 OS = Mus musculus OX = 10090 GN = Rpl9 PE = 2 SV = 2 − [RL9_MOUSE]	Rpl9	165.2937513	80.13333333	2.062734001
Q6ZWY3	40S ribosomal protein S27-like OS = Mus musculus OX = 10090 GN = Rps27l PE = 1 SV = 3 − [RS27L_MOUSE]	Rps27l	251.7	124.87	2.015696324
Q9JHL1	Na(+)/H(+) exchange regulatory cofactor NHE-RF2 OS = Mus musculus OX = 10090 GN = Slc9a3r2 PE = 1 SV = 2 − [NHRF2_MOUSE]	Slc9a3r2	58.31333333	28.93	2.015670008

Immunoprecipitated proteins were identified using liquid chromatography-tandem mass spectroscopy (LC–MS/MS). Forty-eight unique proteins were at least twofold more abundant than in the WT control. Sixteen of these proteins are listed in the table.

Total MPO expression and activity in the lung tissues of WT and RNF128 KO mice following LPS exposure were determined to investigate the specificity of RNF128 for MPO expression and function in the ALI-induced mouse model. LPS significantly increased MPO expression and activity in the lungs of WT and RNF128 KO mice compared to the control group. RNF128 KO mice demonstrated higher MPO expression and activity than WT mice (Fig. 3I–K).

Previous studies have reported that neutrophils are critical for ALI, with PMNs increasing dramatically [30]. The increase in pulmonary neutrophil accumulation allowed us to directly examine the effect of RNF128 on neutrophils from ALI-induced mice. The BALF cells expressed neutrophil markers, including CD11b, MPO, TNF-α, IL-1ß and IL-6. Neutrophils from KO-LPS mice had higher gene expression of inflammatory cytokines than neutrophils from WT-LPS mice. MPO expression was notably elevated in neutrophils from KO-LPS mice (Fig. S4). This finding suggests that RNF128 may regulate MPO expression using methods other than direct protein–protein interactions.

### RNF128 deficiency promoted macrophage activation and neutrophil infiltration

**Fig. 4:**
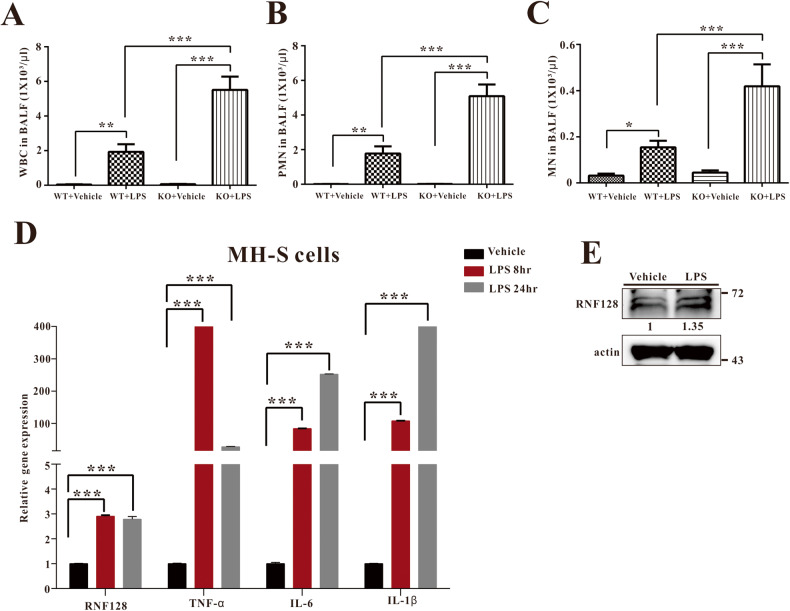
RNF128 deficiency stimulates LPS-induced PMN infiltration and RNF128 expression in the lungs of LPS-treated mice and alveolar macrophages. The total cells (**A**), monocytes (**B**) and PMN (**C**) in the BALF were assessed after LPS injection. **D** The levels of RNF128, TNF-α, IL-6 and IL-1β mRNAs were measured in LPS-stimulated MH-S cells using Q-PCR. **E** RNF128 expression was examined in LPS-stimulated MH-S cells using immunoblotting and the data were quantified using Image J software. The data are presented as mean ± standard deviation (SD). **P < 0.01; ***P < 0.001, Student’s *t*-test.

We profiled immune cell accumulation in the lungs with or without LPS stimulation. Following ALI induction, we observed a significant increase in the WBC count (Fig. 4A). LPS stimulation increased macrophage and neutrophil infiltration in the alveolar space of RNF128 KO mice (Fig. 4B, C).

Under steady-state conditions, alveolar macrophages (90–95% cell content of lung-resident macrophages) maintain the balance between the responses of alveolar epithelial cells, DCs and lung T cells to environmental cues. Alveolar macrophages restrict and repair infection-induced damage to protect against ALI [31]. Here, we propose that RNF128 plays a critical role in the transition of alveolar macrophages from a tolerogenic state to an inflammatory state accompanied by an increase in IL-1β, IL-6 and TNF-α production. LPS treatment increased the expression of RNF128 and inflammatory cytokines in MHS cells, a murine alveolar macrophage. (Fig. 4D, E) The data indicate that RNF128 affects the function of alveolar macrophages and may be implicated in the inflammatory response.

### The effect of RNF128 on alveolar macrophage activation is mediated via the TLR4-NF-κB signalling axis

**Fig. 5:**
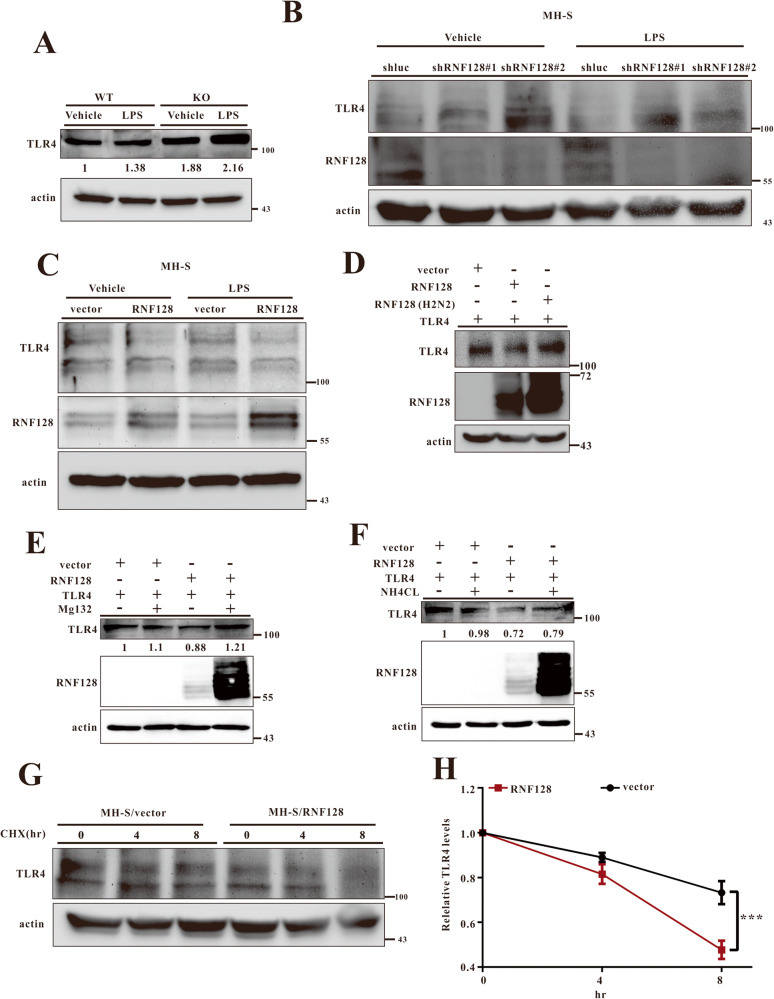
RNF128 reduces TLR4 protein level. **A** Immunoblotting analysis of TLR4 expression in the lung samples from WT and RNF128 KO mice with or without LPS treatment for 24 h. Relative immunoblotting signals were inducted. **B**, **C** Endogenous TLR4 levels in the indicated stable cell lines with or without LPS treatment. **D** TLR4 degradation in cells overexpressing Ring mutant RNF128 or WT cells. HEK293 cells were transfected with 0.5 μg of vector, RNF128, or RNF128 (H2N2) in the presence of 0.5 μg of TLR4. The cells were subsequently harvested and subjected to immunoblotting. **E** HEK293 cells were transfected with 0.5 μg of vector, RNF128, or TLR4 and then treated with Mg132 (50 μM) for 8 h. The cells were subsequently harvested and subjected to immunoblotting and the data quantified using Image J software. (F) HEK293 cells were transfected with 0.5 μg of vector, RNF128 or TLR4 and then treated with NH_4_CL (50 mM) for 8 h. The cells were subsequently harvested and subjected to immunoblotting and the data were quantified using Image J software. **G**, **H** MH-S/vector and MH-S/RNF128 cells were treated with cycloheximide (CHX, 100 μM) and harvested at the indicated times. Cell lysates were subjected to immunoblotting using the indicated antibodies. The data are presented as mean ± SD. The statistical significance was evaluated using the Student’s *t*-test. ***P < 0.001.

Numerous investigations have established that TLR4-mediated signalling regulates alveolar macrophage activation during ALI development [12, 13]. We analysed TLR4 expression levels in the lung tissues of WT and RNF128 KO mice to identify the relationship between RNF128 and TLR4. TLR4 expression levels significantly increased in the lung tissues of RNF128 KO mice (Fig. 5A).

Next, we used MHS cells to determine if RNF128 affected TLR4 expression. TLR4 levels were measured by silencing or overexpressing RNF128 in MHS cells. TLR4 levels increased in RNF128-silenced MHS cells but decreased in RNF128 overexpressed cells (Fig. 5B, C). Overexpressing RNF128 or RNF128 (H2N2) in HEK293 cells confirmed the effect of RNF128 on TLR4 levels. Consistent with the effect of RNF128 on endogenous TLR4, only RNF128 and not RNF128 mutant (H2N2) affected TLR4 levels (Fig. 5D). Next, we used the proteasome degradation inhibitor, Mg132, or the lysosome degradation inhibitor, NH_4_Cl, to determine whether RNF128 degrades TLR4 in proteasomes or lysosomes. The results showed that MG132 but not NH4Cl inhibited the reduction of TLR4 levels induced by RNF128 (Fig. 5E, F). To further determine if RNF128 regulates the stability of endogenous TLR4, we measured the TLR4 levels in the presence of cycloheximide. As a result, the RNF128 affected the TLR4 stability (Fig. 5G, H). Altogether, these results demonstrated that RNF128 reduced TLR4 expression and required E3 ligase activity.

**Fig. 6:**
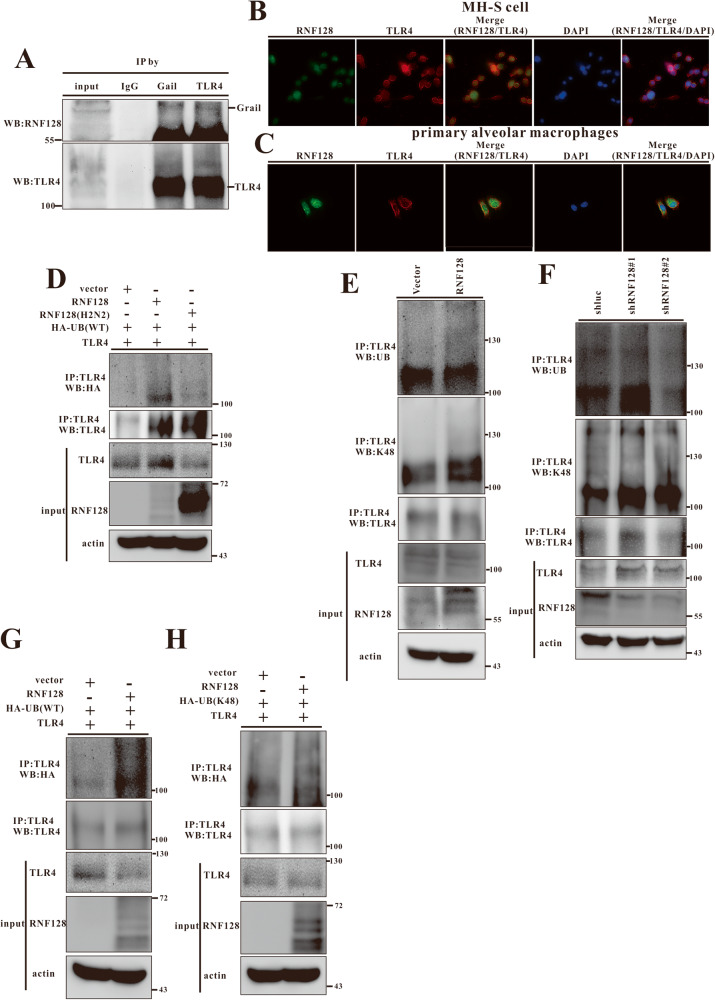
RNF128 interacts with TLR4 and enhances its ubiquitination. **A** Interaction between endogenous RNF128 and TLR4. Extracts from MH-S cells were immunoprecipitated using anti-RNF128, anti-TLR4 or rabbit anti-immunoglobulin G (anti-IgG) antibodies and analysed using anti-RNF128 and anti-TLR4 antibodies. **B**, **C** The subcellular localisation of RNF128 and TLR4 in MH-S or primary alveolar macrophages was examined using immunofluorescence microscopy (THUNDER Imaging Systems). **E**, **F** Lysates from MH-S (RNF128 overexpression or silencing) stable cell lines were extracted, immunoprecipitated (IP) using TLR4 antibody, and analysed for ubiquitylation using the specified antibody. **D**, **G**, **H** HEK293 cells were transiently transfected with HA-Ubiquitin (Ub), HA-Ub-K48, TLR4, RNF128 and RNF128 (H2N2) after 24 h. Harvested lysates were immunoprecipitated using TLR4 antibody and analysed for ubiquitylation after immunoblotting.

**Fig. 7:**
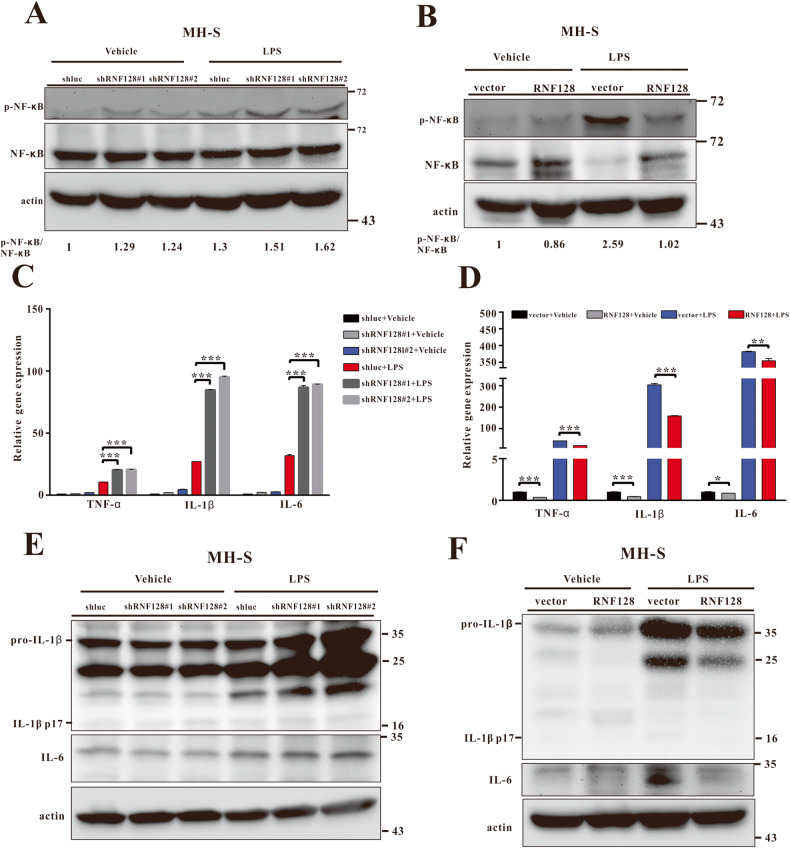
RNF128 suppresses LPS-induced NF-κB activation and pro-inflammatory cytokine expression in MH-S cells. **A** Immunoblots of NF-κB and p-NF-κB in MH-S/shluc and MH-S/shRNF128 cell lines treated with LPS (100 ng/ml) for 2 h. Each lane showed the intensity ratio of NF-κB and p-NF-κB. The results shown are representative of at least three independent experiments. **B** Immunoblots of NF-κB and p-NF-κB in MH-S/vector and MH-S/RNF128 cell lines treated with LPS (100 ng/ml) for 2 h. Each lane showed the intensity ratio of NF-κB and p-NF-κB. The results shown are representative of at least three independent experiments. **C** The expression of TNF-α, IL-1β and IL-6 mRNAs in MH-S/shluc and MH-S/shRNF128 cell lines treated with or without LPS (100 ng/ml) for 2 h. **D** The expression of TNF-α, IL-1β and IL-6 mRNAs in MH-S/vector and MH-S/RNF128 cell lines treated with or without LPS (100 ng/ml) for 2 h. **E** The protein levels of IL-1β, and IL-6 in MH-S/shluc and MH-S/shRNF128 cell lines treated with or without LPS (100 ng/ml) for 2 h. **F** The protein levels of IL-1β, and IL-6 in MH-S/vector and MH-S/RNF128 cell lines treated with or without LPS (100 ng/ml) for 2 h. The data are presented as mean ± SD. The statistical significance was evaluated using one-way ANOVA with Newman–Keuls post-hoc test or Student’s *t*-test. *P < 0.05; **P < 0.01; ***P < 0.001.

The mechanism by which RNF128 regulates TLR4 stability was determined by investigating the interaction between TLR4 and RNF128 using co-immunoprecipitation and immunofluorescence microscopy. We detected a reciprocal interaction between RNF128 and TLR4 in MHS cells (Fig. 6A) and their colocalization in MHS cells and primary alveolar macrophages (Fig. 6B, C). RNF128 is an E3 ubiquitin ligase that enhances target protein ubiquitination. Thus, we hypothesised that RNF128 directly promoted TLR4 ubiquitination and regulated its stability. HEK293 cells were transfected with plasmids encoding RNF128, RNF128 (H2N2), ubiquitin (Ub) and TLR4, and their expressions were measured. Co-expression of RNF128 and not the RING finger domain mutant of RNF128 (H2N2) significantly elevated TLR4 ubiquitination level (Fig. 6D). RNF128 overexpression also enhanced TLR4 ubiquitination in MHS cells compared to the control (Fig. 6E). In contrast, RNF128-knockdown cells exhibited a significant reduction in TLR4 ubiquitination level (Fig. 6F). We compared WT-Ub to mutant Ub containing a single lysine residue (K48) to confirm RNF128-mediated TLR4 ubiquitination. RNF128-mediated TLR4 ubiquitination was significantly increased in the presence of HA-Ub or HA-Ub-K48 (Fig. 6G, H). The observed data revealed that RNF128 mediates the K48-linked ubiquitination of TLR4. LPS triggers the activation of TLR4 and NF-κB, increasing the production of pro-inflammatory cytokines, such as TNF-α, IL-1β and IL-6. We analysed the activation of NF-κB and its downstream targets in RNF128 silenced- or overexpressed- MHS cells to understand how RNF128 affects the TLR4-NF-κB signalling pathway. Indeed, LPS treatment induced NF-κB phosphorylation and inflammatory cytokine expression in MHS/shRNF128 cells more strongly than in controls (Fig. 7A, C, E). RNF128 overexpression reduced the phosphorylation of NF-κB and its downstream targets (Fig. 7B, D, F). These findings demonstrate that RNF128 mediates the activation of alveolar macrophage via the TLR4-NF-κB axis.

### RNF128 overexpression ameliorates lung damage in the LPS-induced ALI mouse model

**Fig. 8:**
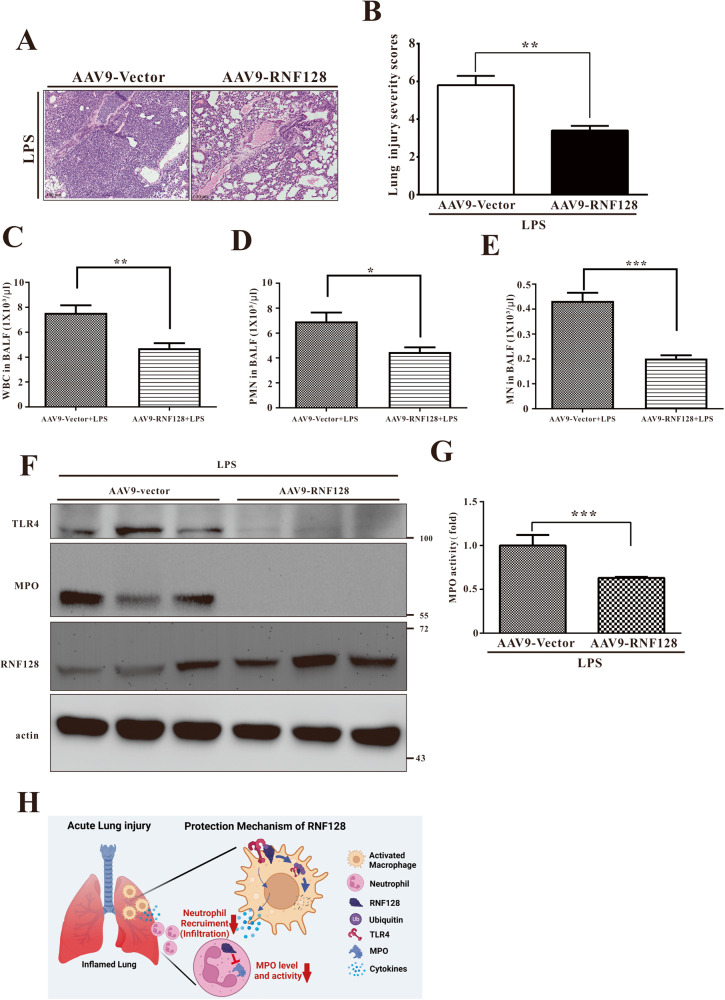
RNF128 overexpression reduces LPS-induced lung injury. **A**, **B** The mice were administered with LPS (i.t.) for 24 h. Histological images of lung tissues stained with haematoxylin and eosin (HE) in the indicated groups. Scale bars: 100 μm. An experienced pathologist assigned severity scores to lung injuries. The total cells (**C**), monocytes (**D**) and PMN (**E**) in the BALF were derived from LPS-stimulated AAV9-Vector and AAV9-RNF128 mice. **F** The protein levels of MPO, TLR4 and RNF128 in the lungs of AAV9-Vector and AAV9-RNF128 mice treated with LPS were detected using immunoblotting. **G** MPO activity was determined in the lung tissues from the indicated groups. **H** The working model of RNF128 in the regulation of LPS-induced ALI. The data are presented as mean ± standard deviation (SD). The statistical significance was evaluated using one-way ANOVA with Newman–Keuls post hoc test or Student’s *t*-test. *P < 0.05; **P < 0.01; ***P < 0.001.

RNF128 improved LPS-induced ALI by regulating MPO expression and activity, alveolar macrophage activation and neutrophil infiltration. We used AAV9-mediated RNF128 overexpression in the lungs via intranasal delivery to investigate the function of added RNF128 in developing LPS-induced ALI in mice. RNF128 expression was dramatically elevated in lung tissues of LPS-induced mice treated with AAV9-Vector or AAV9-RNF128 (Fig. 8F and Fig. S5).

In contrast to RNF128 KO mice, LPS-treated AAV9-RNF128 mice had significantly reduced alveolar septal thickness, peribronchial and perivascular cuffing, congestive capillaries and PMN infiltration in the lungs (Fig. 8A, B). Consistently, AAV9-RNF128 mice exhibited a significant reduction in the total number of macrophages and neutrophils in BALF than the controls (Fig. 8C–E). We analysed MPO expression and activity following RNF128 overexpression because this gene regulates MPO expression. As expected, LPS-treated AAV9-RNF128 mice had significantly lower MPO levels and activity than the controls (Fig. 8F, G). Consistently, TLR4 level was lower in the lung of LPS-treated AAV9-RNF128 mice than in the controls (Fig. 8F). These findings suggest that RNF128 protects lung tissue and improves lung damage following LPS-induced ALI in mice.

## Discussion

Acute respiratory failure, severe inflammatory response, and high mortality and morbidity are hallmarks of ALI and ARDS [26]. Therefore, discovering novel molecular regulatory mechanisms is crucial for optimising treatment and preventing ALI. The LPS-induced ALI mice model is widely used in the pathophysiological studies of ALI/ARDS [32]. Several studies have demonstrated that the mouse strains are responsible for the susceptibility in the progression of LPS-induced ALI [33, 34]. The inbred mouse strains of the C57BL/6 mice were found to be more susceptible to acute lung inflammation, injury and leucocyte infiltration [35]. Besides, the C57BL/6 has been extensively used for genetically engineered mice [36]. Therefore, we have used the C57BL/6 mice in further studies.

In this study, RNF128 knockout caused significant deterioration of the lungs, increased cytokine expression and immune cell infiltration in LPS-administered mice. Furthermore, RNF128 overexpression significantly attenuated LPS-induced lung injury and inflammatory response. Mechanistically, RNF128 reduced the activation of alveolar macrophages and neutrophils by inhibiting TLR4 and MPO, respectively. Together, these findings suggest that lung tissue RNF128 prevents LPS-induced lung injury, immune cell infiltration and inflammation, making it a potential therapeutic target in treating ALI (Fig. 8H).

Previous studies have linked the NLRP3 inflammasome, a multiprotein complex involved in cellular inflammation, to the onset of ALI. The activation of the NLRP3 inflammasome in response to a stimulus, stimulates IL-1β maturation and secretion via active caspase-1. Alveolar macrophages are the major lung-resident macrophages and the primary sources of IL-1β in ALI [37–39]. In this study, we report that LPS administration to RNF128 KO mice significantly increased lung IL-1β levels. In contrast, LPS-treated AAV9-RNF128 mice showed a reduction in IL-1β levels. Similar results were observed for alveolar macrophages. Therefore, additional research is required to comprehend the relationship between RNF128 and NLRP3 and the diverse molecular pathways implicated in ALI development.

This is the first study demonstrating that endogenous RNF128 modulates TLR4 protein expression by regulating TLR4 ubiquitination. TLR4 is an attractive therapeutic target, and TLR4‐targeting drugs have been extensively evaluated in clinical trials [40]. TLR4 agonists, such as lipid A mimics, have contributed to developing more specific and effective vaccine adjuvants [41]. In contrast, TLR4 antagonists prevent cytokine production at an early stage [42]. LPS-induced RNF128 expression suppresses TLR4-signalling and downstream pro-inflammatory cytokine generation. RNF128 overexpression may be a novel therapeutic strategy for inflammation-related diseases. Indeed, overexpression of RNF128 in the lungs of mice with LPS-induced ALI successfully protected lung tissue damage. Importantly, TLR4 is critically involved in LPS- and virus-induced ALI [13], including COVID-19-induced ALI [43]. It would be interesting to determine whether RNF128 expression is elevated in COVID-19 patients and whether RNF128 overexpression attenuates COVID-19-induced inflammation.

This study demonstrated that RNF128 in alveolar macrophages plays a significant role in LPS-induced ALI. RNF128 reduces the LPS-induced inflammatory response and damage in the lungs. TLR4 and its downstream signalling pathways, including the NF-κB pathway, mediate the LPS-induced inflammatory response [44]. This study revealed that RNF128 interacts with TLR4 to promote its ubiquitination and degradation, reducing the LPS-induced inflammatory response. However, we could not determine whether RNF128 specifically interacted with TLR4. Moreover, RNF128 may target other molecules in LPS-activated macrophages besides TLR4.

ALI is a condition of acute inflammation that disrupts the endothelial and epithelial barriers of the lungs. ALI is characterised by a cascade of cellular responses, including alveolar-capillary membrane disruption, macrophage activation leading to the release of pro-inflammatory cytokines, and excessive neutrophil migration to the alveoli. Excessive neutrophils disrupt the basement membrane, increasing the permeability of the alveolar-capillary barrier and releasing cytotoxic mediators such as elastase, which degrade epithelial junction proteins and damage the epithelium [26, 45]. Neutrophils are the most prevalent cell type in the innate immune system. They are crucial in host defence against pathogens such as bacteria and fungi [6]. First, neutrophils generate superoxide anions via nicotinamide adenine dinucleotide phosphate (NADPH) oxidase activity. Reactive oxygen species, such as superoxide radicals, hydrogen peroxide, hypochlorous acid, hydroxyl radicals and chloramines, are generated in response to pathogen stimulation. Neutrophils mostly release MPO, a critical mediator of anti-pathogenic response [46–48]. In this study, we observed that RNF128 affected macrophages and neutrophils through distinct mechanisms. First, RNF128 reduces macrophage inflammation by modulating TLR4 stability and signalling pathway activation. Simultaneously, the PMN infiltration decreased. Here, we demonstrate that RNF128 protects against LPS-induced ALI. We also identified RNF128-associated proteins using mass spectrometry to clarify their role in ALI. Based on these results, we identified several RNF128-interacting proteins in LPS-treated lung tissues (Table 1). Interestingly, the protein expression and enzyme activity of the RNF128-interacting protein, MPO, are elevated in RNF128 KO mice. In contrast, MPO protein levels and activity were reduced in AAV9-RNF128 mice. Similarly, the interaction between RNF128 and MPO was confirmed in differentiated HL-60 cells. We also found that RNF128 regulated MPO expression. Recent research indicates that MPO deficiency improves most inflammatory diseases, including pulmonary inflammation, cardiovascular disease and metabolic syndrome [49]. Therefore, regulating RNF128 expression or developing peptides to attenuate MPO activity may be effective treatment approaches for MPO-induced organ or tissue damage.

In conclusion, our study identified RNF128 as a potential ALI regulator. The underlying mechanism of this regulation has also been elucidated. We believe our findings will serve as an excellent platform for developing novel ALI treatment strategies.

## Methods

### Animal experiments

RNF128 KO mice were generated using the Transgenic Mouse Models Core (Taipei, Taiwan) using CRISPR-Cas9 technology, and KO mice were generated on a C57BL/6J background as per a previous study. The mice were housed under a regular 12 h light/dark cycle for two weeks before the experiments. Eight-week-old male mice were treated with intranasal LPS instillation (2 μg/g body weight). The experiment was terminated 24 h after LPS inhalation, and lung tissues, BALF, primary neutrophils and primary alveolar macrophages were harvested and collected for further analysis.

### Cells, plasmids and transfection procedures

MH-S cells were cultured for 24 h in RPMI-1640 medium containing 10% foetal bovine serum (FBS) plus 0.05 mM 2-mercaptoethanol. HL-60 cells were cultured in RPMI with 10% FBS in 5% CO_2_-humidified air at 37 °C. To differentiate HL-60 cells into granulocyte-like cells, the cells were incubated for six days with DMSO (1.3%). For further experiments, the cells were maintained in RPMI. The RNF128 gene was cloned into the pCMV-TNT vector (Promega, CA, USA) using the EcoRI and BamHI sites. The p3xFLAG-CMV-TLR4 plasmid was purchased from Addgene (Watertown, MA). Transfection was performed using Fugene 6 (Roche, Basel, Switzerland) according to the manufacturer’s instructions. Cells were plated and allowed to grow to 70–80% confluence, followed by Fugene 6-mediated gene transfection. Transfected cells were harvested after 48 h and lysed in RIPA buffer (100 mM Tris-HCl pH 8.0, 150 mM NaCl, 0.1% sodium dodecyl sulfate (SDS) and 1% Triton X-100).

### Isolation and culture of primary alveolar macrophages

Primary alveolar macrophages were isolated from the BALF of 6-week-old C57BL/6J WT and RNF128 KO mice, and subsequently cultured. Briefly, the mice were anaesthetized, the lungs were perfused three times with 1 ml of PBS, and the BAL fluid was retrieved. For each experiment, BAL fluid from three to five mice was pooled and centrifuged at 300×*g* for 10 min, and the pellet was resuspended in Dulbecco’s Modified Eagle Medium containing 10% FBS. The cells were plated on a 6-well plate and incubated at 37 °C overnight.

### Immunoprecipitation and immunoblot analysis

Cells were harvested using the lysis buffer (50 mM Tris pH 8.0, 5 mM NaCl, 0.5% NP-40 and 1× protease inhibitor), freeze/thawed three times, and the proteins were recovered. The protein concentration was determined using the Bradford method (Bio-Rad, CA, USA). Cell extracts containing equivalent amounts of proteins were immunoprecipitated in lysis buffer containing the polyclonal antibody against RNF128, TLR4 or MPO at 4 °C overnight. Dynabeads™ Protein G (Invitrogen, Waltham, MA, USA) were added to the immunoprecipitation mixture for 1 h before three washes with SNNTE buffer (5% sucrose, 1% NP-40, 0.5 M NaCl, 50 mM Tris pH 7.4 and 5 mM EDTA). The entire precipitate was then suspended in the SDS-PAGE sample buffer, boiled and loaded onto an SDS-polyacrylamide gel. The separated proteins were transferred to a nitrocellulose membrane, and the blot was probed with the indicated primary antibodies followed by a secondary antibody (horseradish peroxidase-conjugated anti-mouse or anti-rabbit IgG in PBS/Tween 20 with 5% Carnation nonfat milk). Proteins were detected using enhanced chemiluminescence reagents (GE Healthcare, Chicago, IL, USA). The primary antibodies used for immunoblotting were: anti-TLR4 (19811-1-AP; Proteintech, Rosemount, IL, USA), anti-ubiquitin (P4D1; Cell Signalling, USA), anti-K48 ubiquitin (D9D5; Cell Signalling, Danvers, MA, USA), anti-NF-κB (8242; Cell Signalling, USA), anti-p-NF-κB (3033; Cell Signalling, USA), anti-actin (MAb1501; Chemicon, Rolling Meadows, IL, USA), anti-IL-6 (21865-1-AP; Proteintech, USA), anti-IL-6 (E-AB-70050; Elabscience, Houston, TX, USA), anti-IL-1β (GTX74034; GeneTex, Alton Pkwy Irvine, CA, USA) and anti-RNF128 (The RNF128 antibody is made in our laboratory).

### In vivo ubiquitination assays

The MH-S/RNF128 or MH-S/shRNF128 cells were lysed in the lysis buffer (50 mM Tris pH 8.0, 5 mM NaCl, 0.5% NP-40 and 1× protease inhibitor), freeze/thawed three times, and the proteins were recovered. Cell extracts containing equivalent amounts of protein were immunoprecipitated overnight at 4 °C in the lysis buffer containing anti-TLR4 antibody. Dynabeads Protein G (Invitrogen) was added to the immunoprecipitation mixture and incubated at 4 °C for 1 h. Thereafter, the samples were washed thrice with SNNTE buffer (5% sucrose, 1% NP-40, 0.5 M NaCl, 50 mM Tris, pH 7.4 and 5 mM EDTA). The precipitates were then resuspended in the SDS-PAGE sample buffer and loaded onto the SDS-polyacrylamide gel. After electrophoresis, the gel was transferred onto a nitrocellulose membrane, and the blot was probed with the indicated primary antibodies. Proteins of interest were detected using enhanced chemiluminescence reagents (GE Healthcare).

### Cytokine detection

The concentrations of TNF-α (MTA00B; R&D Systems, USA), IL-6 (M6000B; R&D Systems, USA), and MCP-1 (MJE00B; R&D Systems, USA) in BALF were detected using ELISA kits, according to the manufacturer’s instructions (R&D Systems).

### Measurement of MPO activity

Lung tissues were collected and homogenised in PBS containing 0.5% HTAB. After centrifugation, the supernatant was diluted with the reaction solution. MPO activity was measured using an MPO chlorination activity assay kit (STA-803, CA, USA). Changes in optical density were measured at 410 nm to calculate the MPO activity.

### Determination of the cell counts and protein content in the BALF

The lungs were washed three times with 1× ice-cold PBS. The fluid was centrifuged at 1000 rpm for 5 min at 4 °C, and the supernatant was then collected for further analysis. The cell pellets were resuspended in PBS (0.5 mL) and counted with a hemocytometer. The total protein concentration in the BALF was determined using a BCA protein assay kit (Pierce, Rockford, IL, USA).

### Virus particle production, viral transduction and RNA interference

RNF128 was cloned into vector pQCXIP (Clontech Laboratories, Mount View, CA, USA). The pQCXIP-RNF128 and pQCXIP-empty plasmids were transfected into GP2-293 cells using jetPRIME® (Polyplus, NY, USA). The shRNA oligonucleotides were cloned into the expression vector, pSIREN-Retro-Q (Clontech Laboratories). The retroviruses were prepared according to the protocol available on the Clontech website. (RNF128 shRNA target sequence 1: 5′-GAGGCATCCAAGTCACAATGG-3′; RNF128 shRNA target sequence 2: 5′-GCAGGAAGCAGAGGCAGTTAA-3′). Cells were infected with the indicated retroviruses in the selection medium containing 2 μg/mL polybrene. After 48 h of infection, the cells were treated with 2 μg/mL puromycin to select puromycin-resistant clones. The AAV expression vector was constructed and manipulated using the Helper Free Expression System (Cell Biolabs, Inc., San Diego, CA, USA). RNF128 was cloned into the AAV expression vector, pAAV-MCS. AAV9 overexpressing RNF128 was generated according to the standard protocols.

### Quantitative reverse-transcription PCR

The RNA from cells and tissues was isolated using the TRIZol reagent (Sigma, St. Louis, MO, USA). Complementary DNAs were synthesised using Epicentre MMLV. The gene expressions in cells were analysed using the Applied Biosystems 7500 Real-Time PCR System and the IQ2 FAST qPCR kit. The Gene expressions of tissues were examined using the Roche LightCycler 480. The primers used are listed in Supplementary Table 2.

### Immunohistochemistry

The lung tissues were fixed with 4% formaldehyde overnight at room temperature and embedded in paraffin. The sections were mounted on glass slides, deparaffinized using xylene and stained with RNF128 antibody or by HE staining.

### Immunofluorescence staining

The cells were grown on glass coverslips. Cells were later fixed in 4% paraformaldehyde for 10 min and permeabilized using 0.1% Triton X-100 for 10 min. The slides were washed three times with PBS and blocked with 2% bovine serum albumin (BSA) in PBS for 30 min, and incubated with the primary antibody rabbit polyclonal anti-RNF128 or mouse monoclonal anti-TLR4 (sc-293072; Santa Cruz, USA) overnight at 4 °C in 1% BSA in PBS, and later with the secondary Alexa-Fluor 488 or Alexa-Fluor 594 antibody (Invitrogen) for 1 h in 1% BSA in PBS at 37 °C, and the nuclei were stained with DAPI for 10 min. Cells were mounted with vectashield (Vector Laboratories, Burlingame, CA, USA), and images were examined using immunofluorescence microscopy (THUNDER Imaging Systems).

### Study approval

All animal experiments were approved by the National Defence Medical Centre Animal Experiment Ethics Committee (IACUC-21-192).

### Statistical analysis

The graphing and statistical analysis of the data was performed using GraphPad Prism 7 (GraphPad Software). All data were expressed as mean ± standard deviation. For comparing multiple data sets, one-way analysis of variance (ANOVA) with multiple comparative analyses was used. For analysing two data sets, an unpaired two-tailed Student’s *t*-test was used. *P* < 0.05 was considered statistically significant.

## Supplementary information

Original Data File

Supplementary Information

Supplementary Figure 1

Supplementary Figure 2

Supplementary Figure 3

Supplementary Figure 4

Supplementary Figure 5

Reproducibility checklist

## Data Availability

The experimental data sets generated and/or analysed during the current study are available from the corresponding author upon reasonable request. No applicable resources were generated during the current study.
