# Supplementary material for: RNF128 regulates neutrophil infiltration and myeloperoxidase functions to prevent acute lung injury
Source: Cell Death Dis. 2023 Jun 21;14(6):369. doi: 10.1038/s41419-023-05890-1 (PMC10284794; doi:10.1038/s41419-023-05890-1)
Supplement: Supplementary file 1 — Original Data File [file 41419_2023_5890_MOESM1_ESM.pdf]

Figure 1

Figure 1B

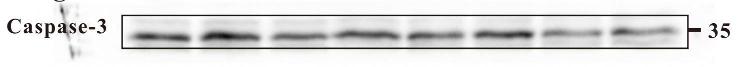

Figure 1B

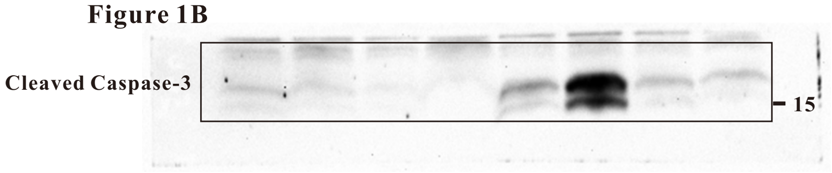

Figure 1B

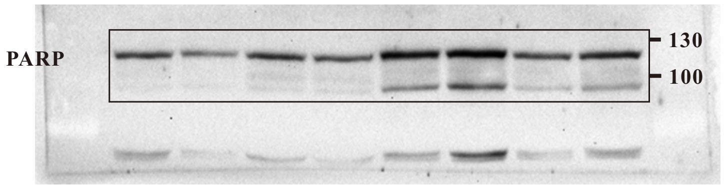

Figure 1B

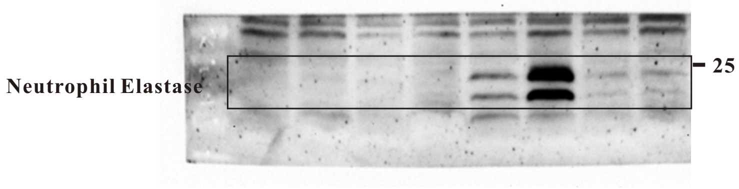

Figure 1B

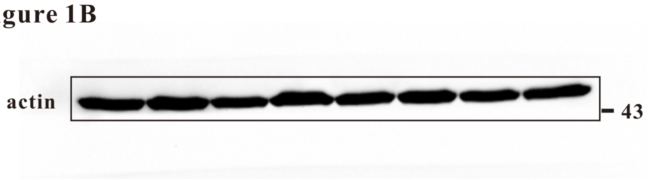

Figure 1D

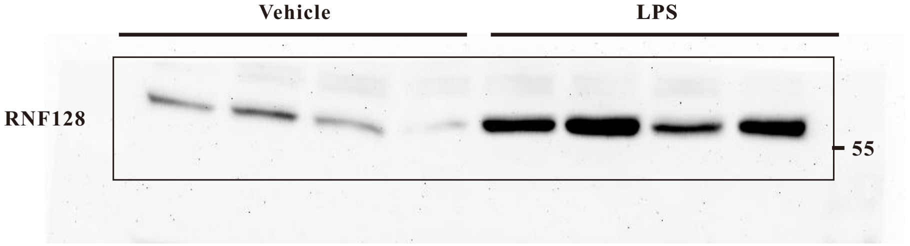

Figure 1D

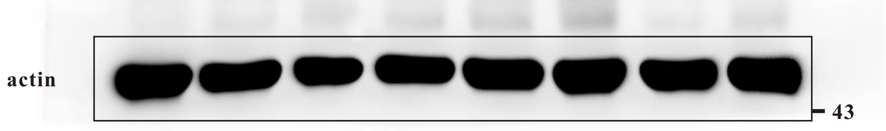

Figure 2

H

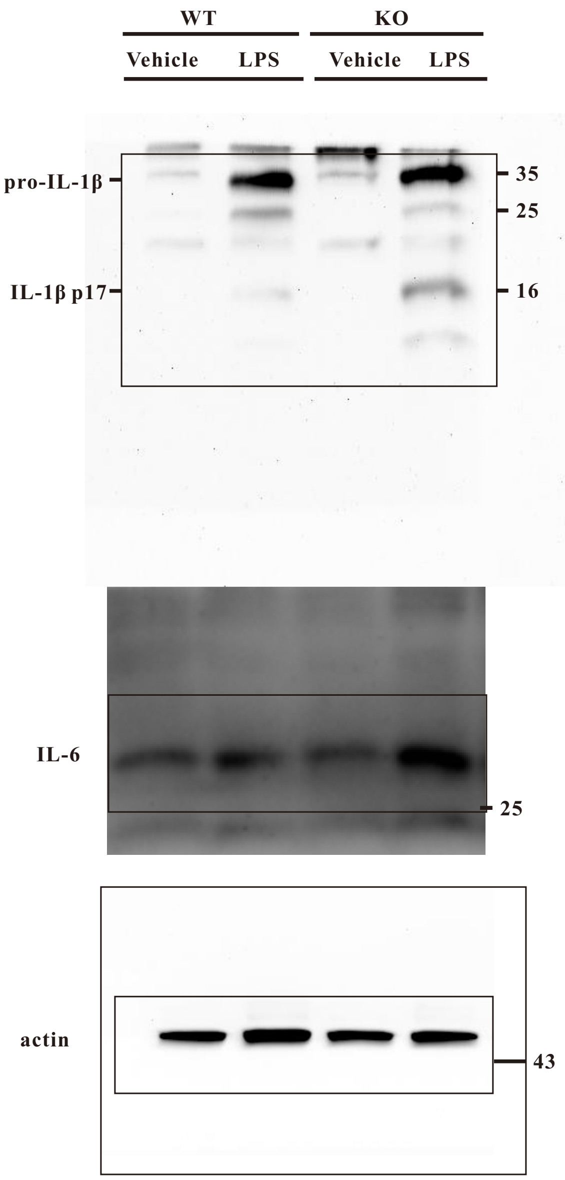
WT
KO
Vehicle
LPS
Vehicle
LPS
pro-IL-1β
IL-1β p17
35
25
16
IL-6
25
actin
43

Fig. 3A

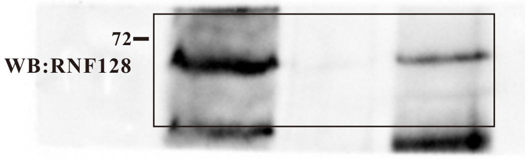

Fig. 3A

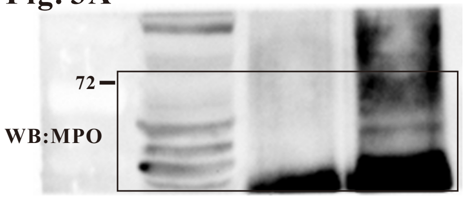

Fig. 3C

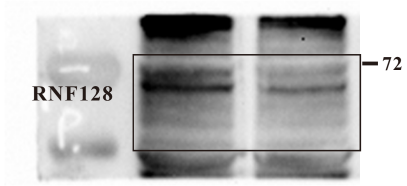

Fig. 3C

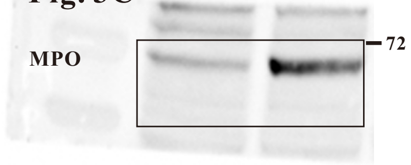

Fig. 3C

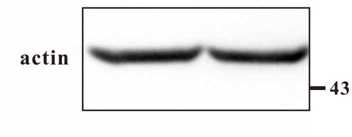

Fig. 3B

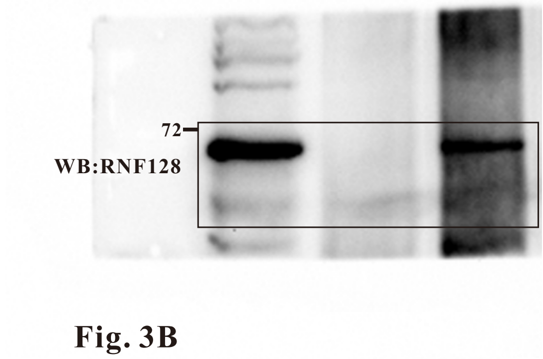

Fig. 3B

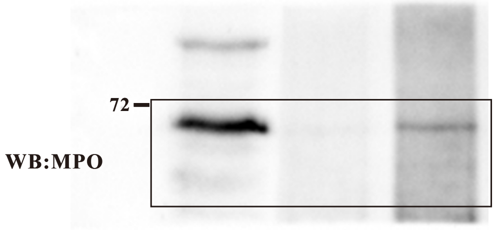

Fig. 3D

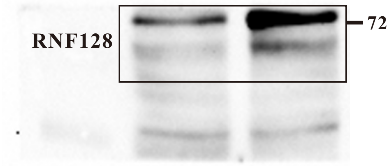

Fig. 3D

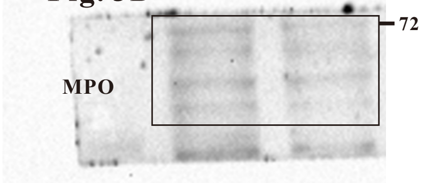

Fig. 3D

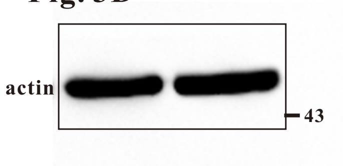

Fig. 3E

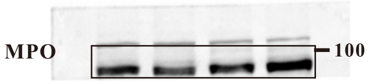

Fig. 3E

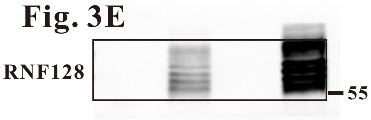

Fig. 3E

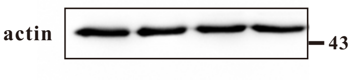

Fig. 3F

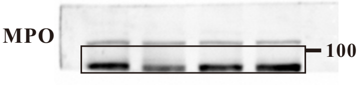

Fig. 3F

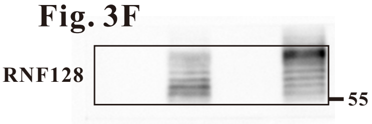

Fig. 3F

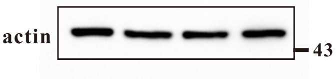

Fig. 3G

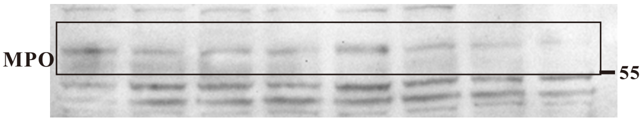

Fig. 3G

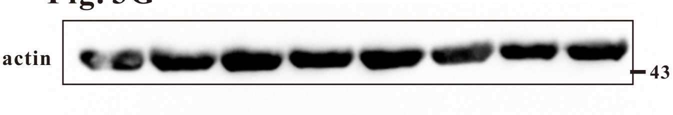

Fig. 3I

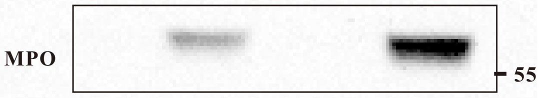

Fig. 3I

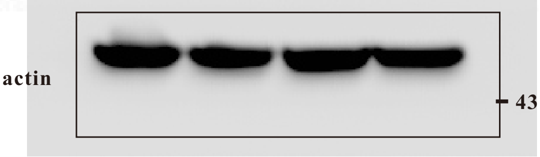

Figure 4

E

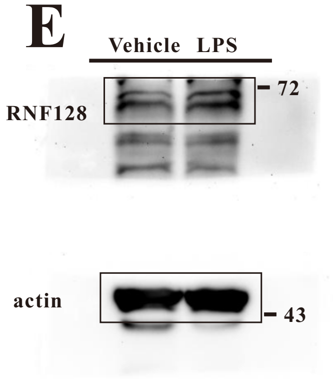

Fig. 5A

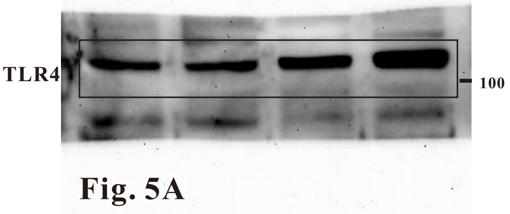

Fig. 5A

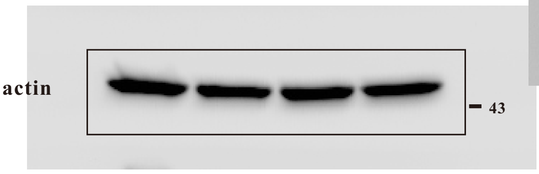

Fig. 5B

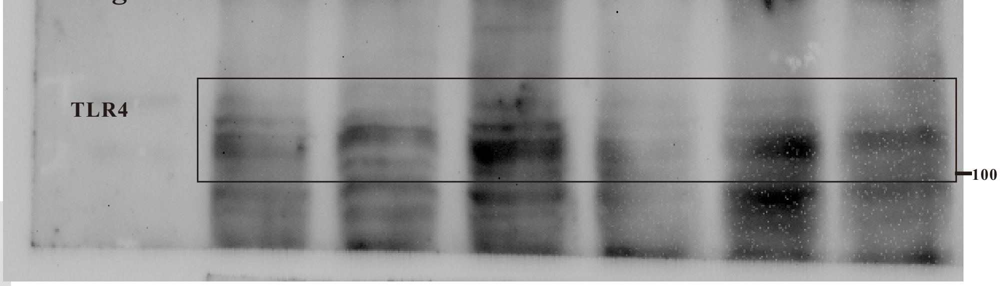

Fig. 5B

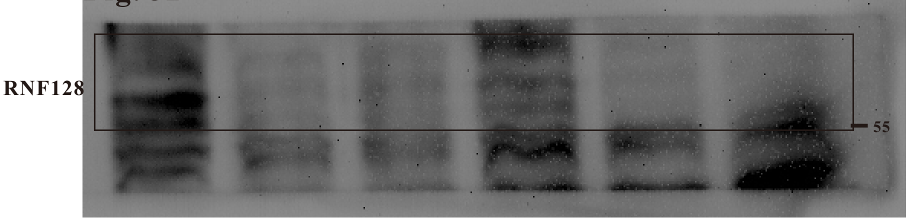

Fig. 5B

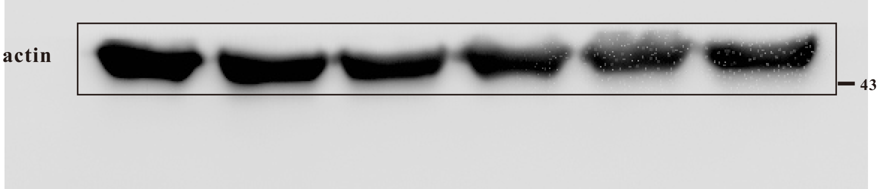

Fig. 5C

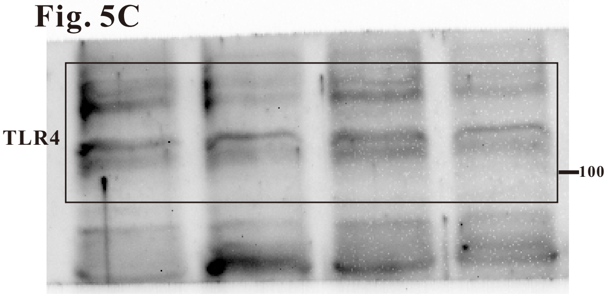

Fig. 5C

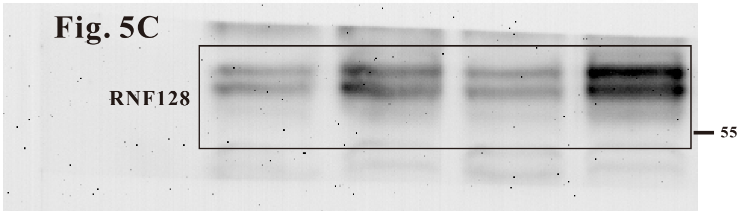

Fig. 5C

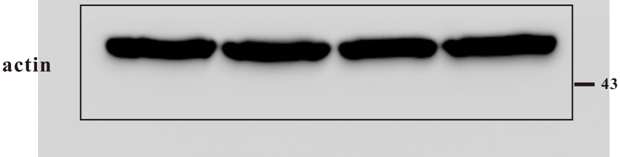

Fig. 5D

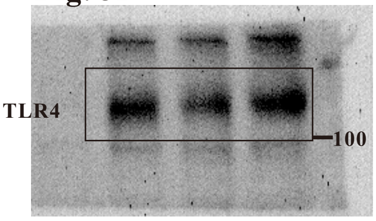

Fig. 5D

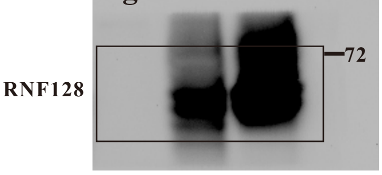

Fig. 5D

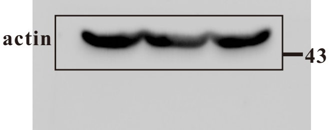

Fig. 5G

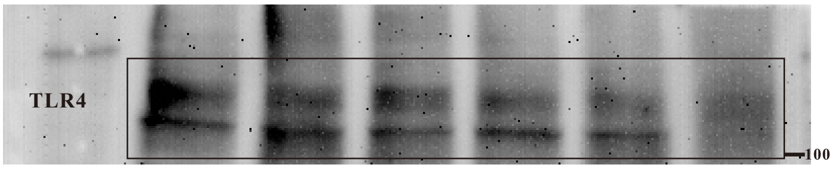

Fig. 5G

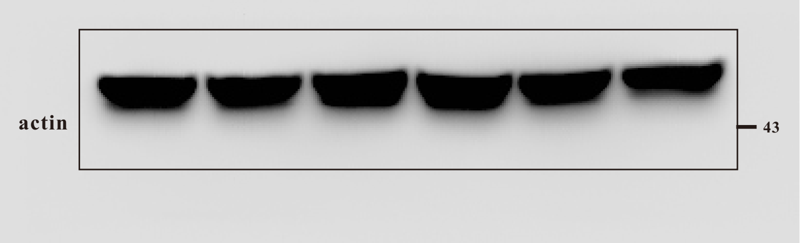

Fig. 5E

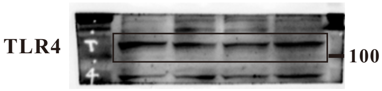

Fig. 5E

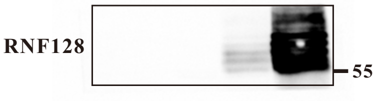

Fig. 5E

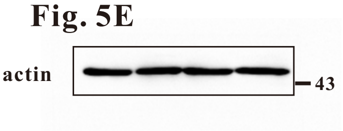

Fig. 5F

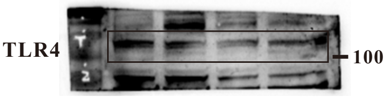

Fig. 5F

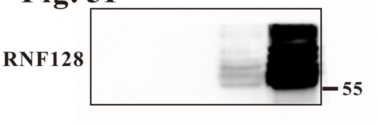

Fig. 5F

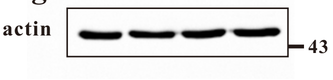

Fig. 6A

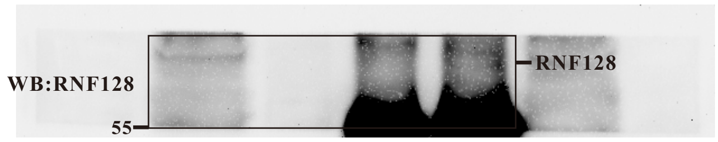

Fig. 6A

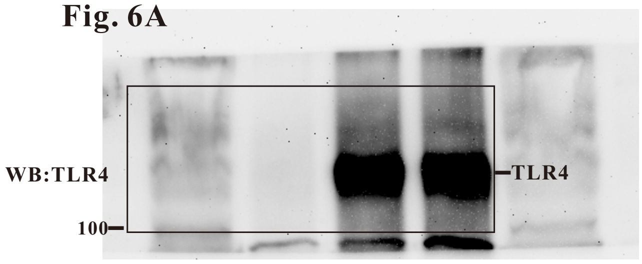

Fig. 6D

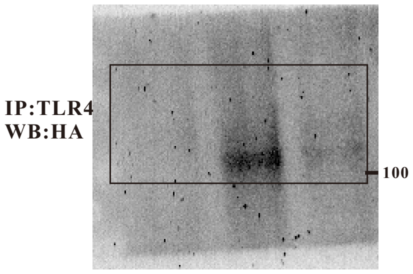

Fig. 6D

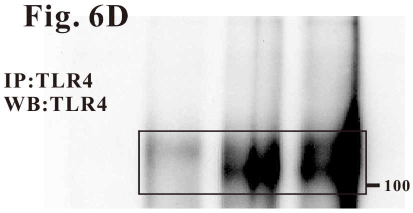

Fig. 6D

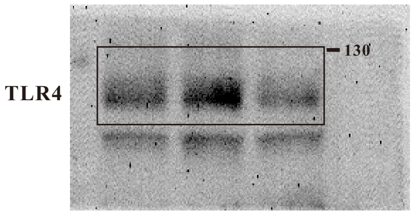

Fig. 6D

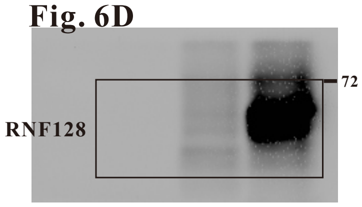

Fig. 6D

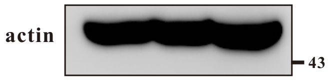

Fig. 6E

IP:TLR4
WB:UB

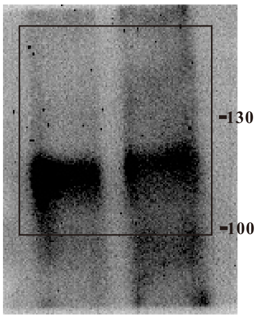

Fig. 6E

IP:TLR4
WB:K48

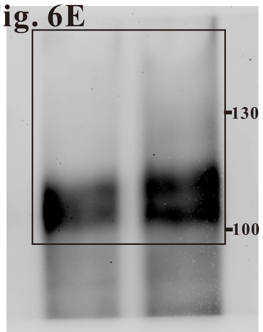

Fig. 6E

IP:TLR4
WB:TLR4

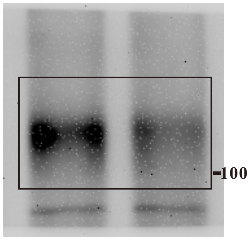

Fig. 6E

TLR4

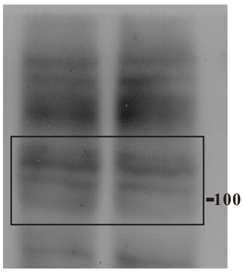

Fig. 6E

RNF128

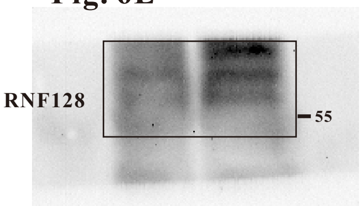

Fig. 6E

actin

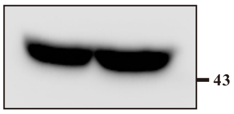

Fig. 6F

IP:TLR4
WB:UB

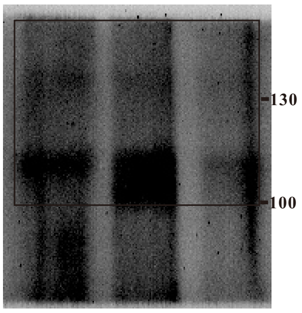

Fig. 6F

IP:TLR4
WB:K48

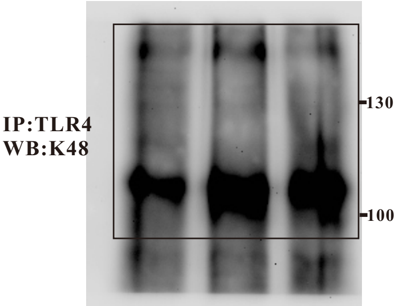

Fig. 6F

IP:TLR4
WB:TLR4

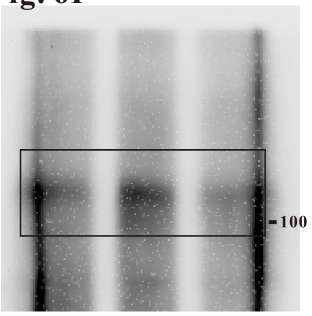

Fig. 6F

TLR4

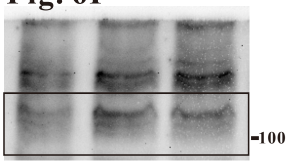

Fig. 6F

RNF128

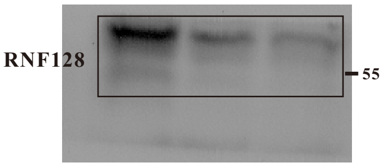

Fig. 6F

actin

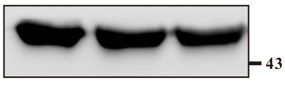

Fig. 6G

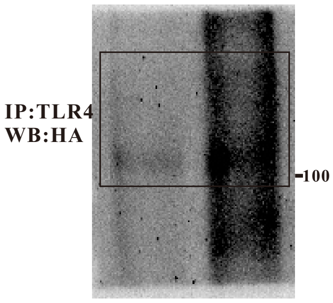

Fig. 6G

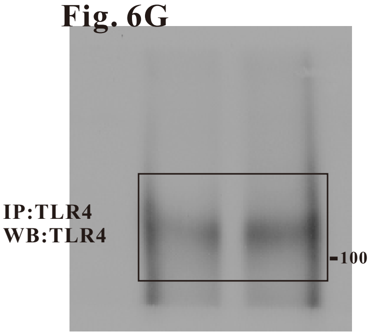

Fig. 6G

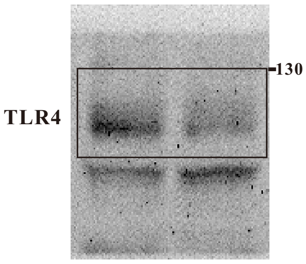

Fig. 6G

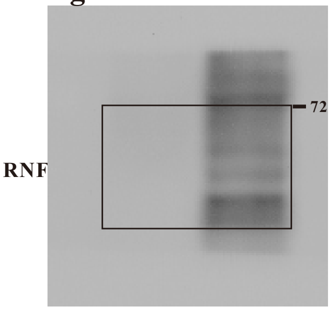

Fig. 6G

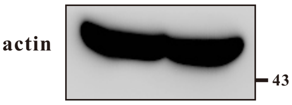

Fig. 6H

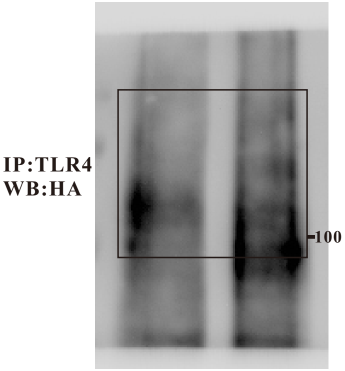

Fig. 6H

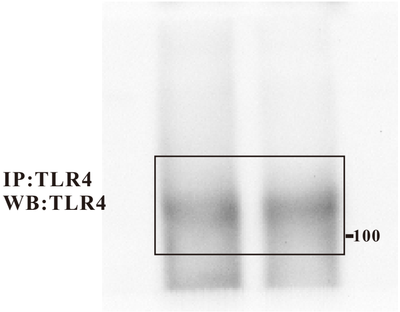

Fig. 6H

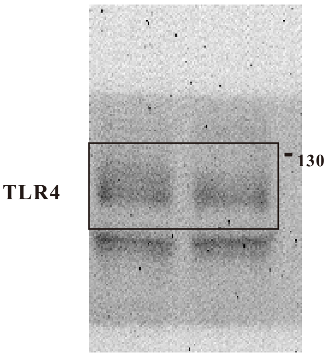

Fig. 6H

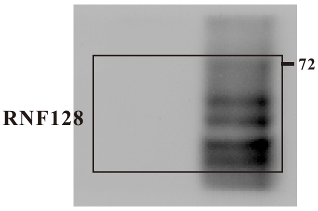

Fig. 6H

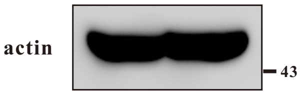

Fig. 7A

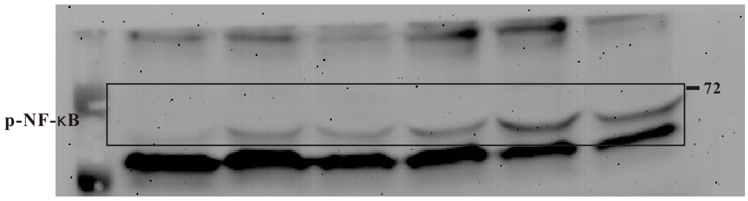

Fig. 7A

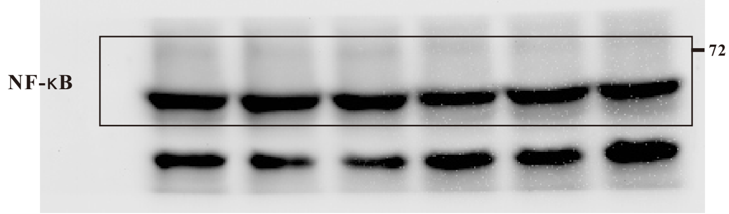

Fig. 7A

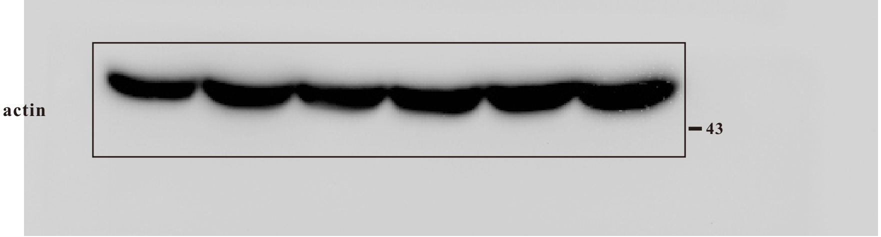

Fig. 7F

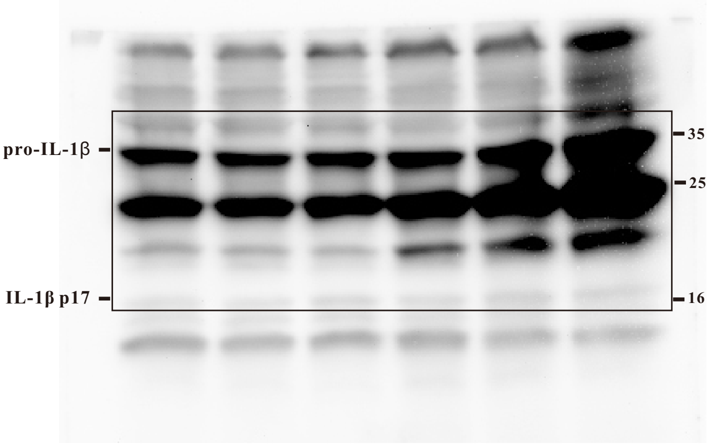

Fig. 7F

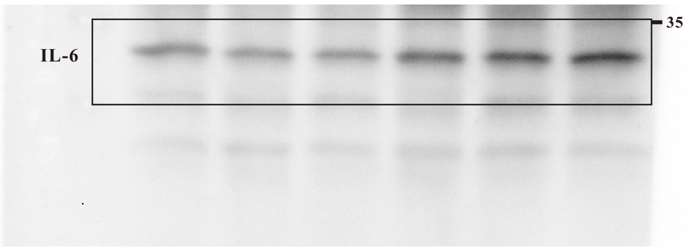

Fig. 7F

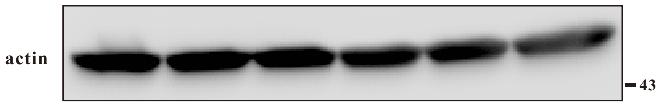

Fig. 7C

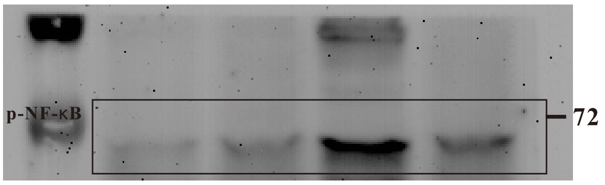

Fig. 7C

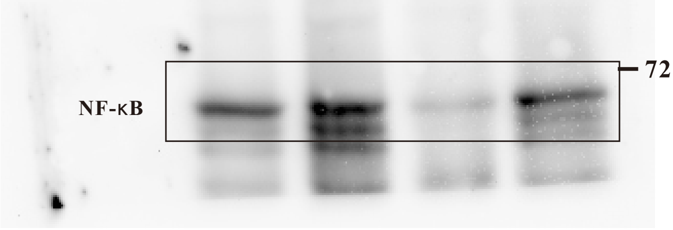

Fig. 7C

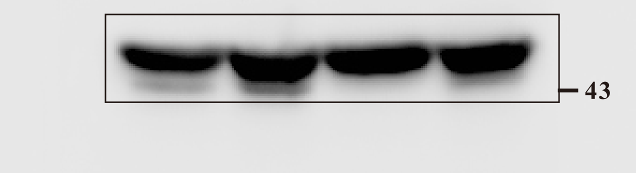

Fig. 7H

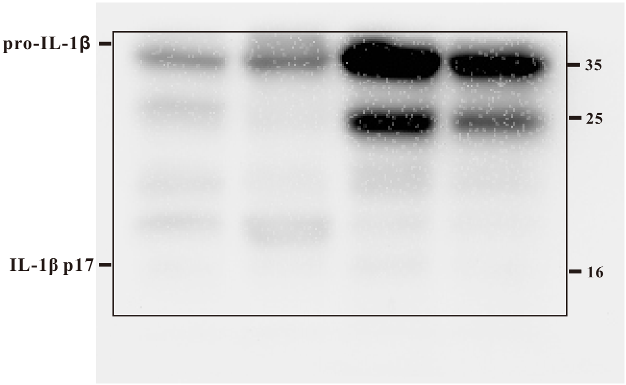

Fig. 7H

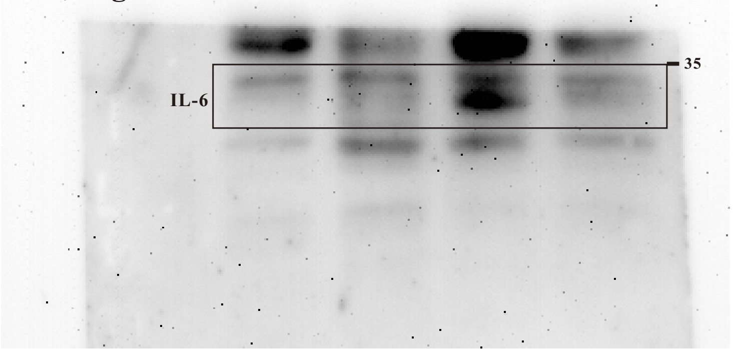

Fig. 7H

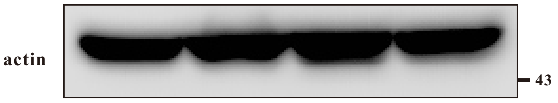

Fig. 8F

MPO

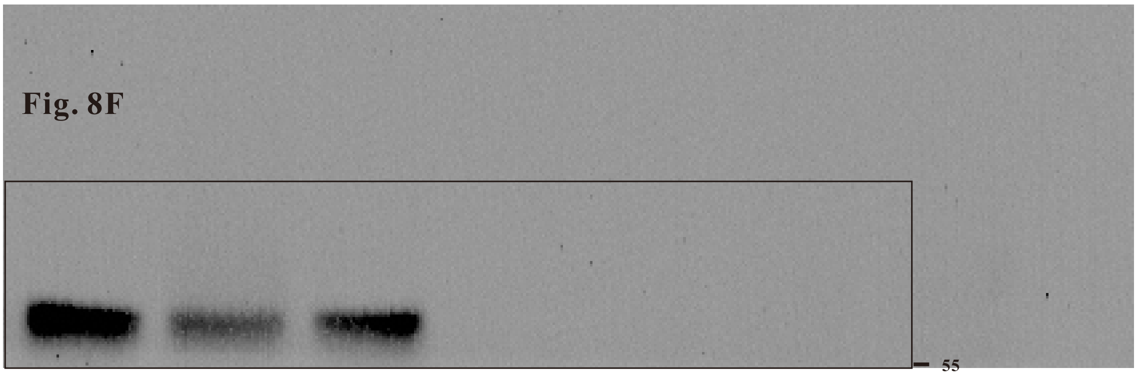

55

Fig. 8F

TLR4

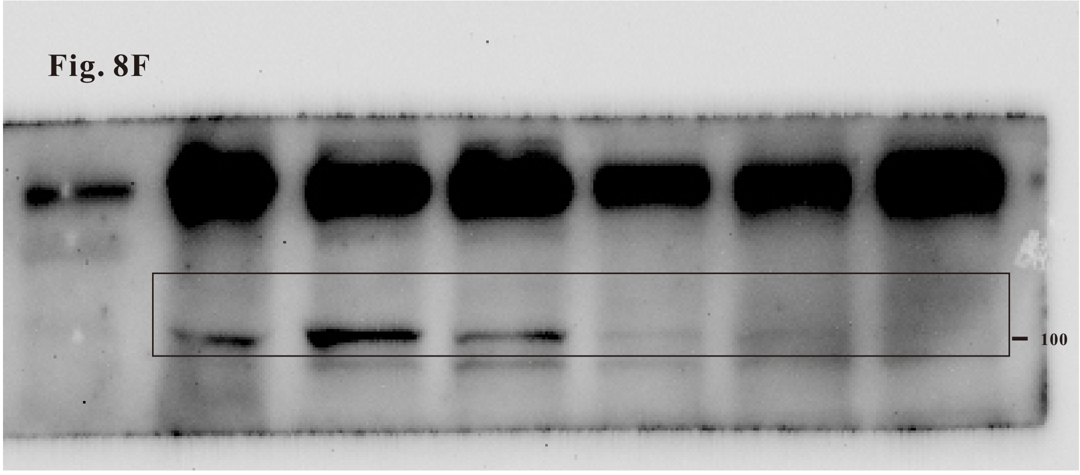

100

Fig. 8F

RNF128

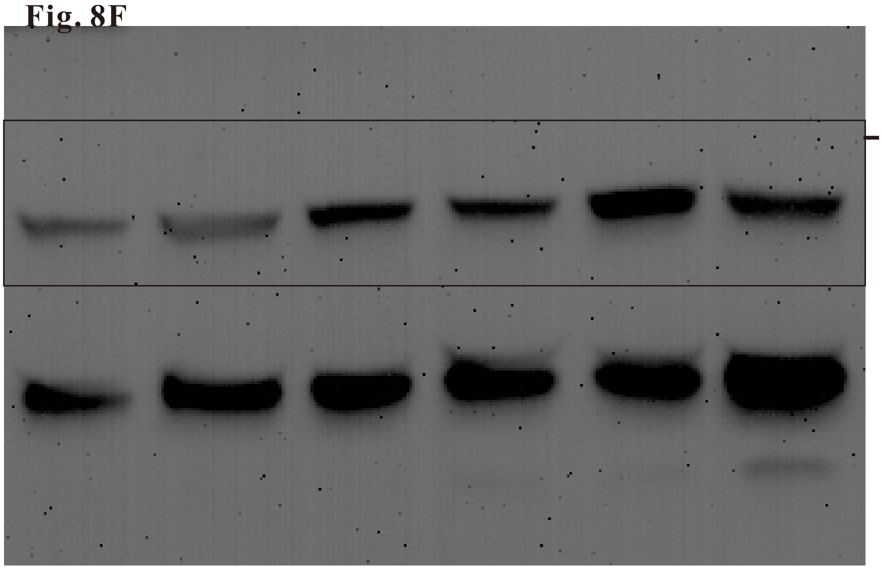

72

Fig. 8F

actin

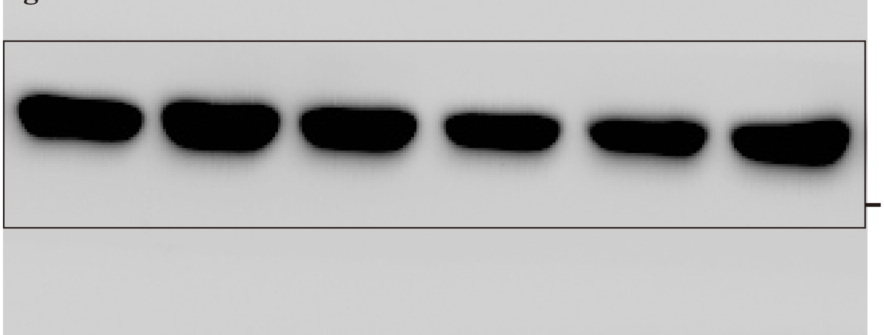

55
