# Supplementary material for: RNF128 regulates neutrophil infiltration and myeloperoxidase functions to prevent acute lung injury
Source: Cell Death Dis. 2023 Jun 21;14(6):369. doi: 10.1038/s41419-023-05890-1 (PMC10284794; doi:10.1038/s41419-023-05890-1)
Supplement: Supplementary file 2 — Supplementary Information [file 41419_2023_5890_MOESM2_ESM.docx]

**Supplementary Information**

**Supplementary Figure Legends**

**Supplementary Fig. 1 RNF128 expression is upregulated in the lungs of LPS-treated mice.** Histological images of lung sections stained using RNF128 antibody (scale bar, 50 μm).

**Supplementary Fig. 2 RNF128 improved ALI-induced inflammatory response.** **A-D** The levels of TNF-α, IL-6, MCP-1 and IL-1β proteins in BALF were detected using ELISA. The statistical significance was evaluated using one-way ANOVA with Newman–Keuls post-hoc test or Student’s t-test. *P < 0.05; **P < 0.01; ***P < 0.001.

**Supplementary Fig. 3 Diagram of the immunoprecipitation-mass spectrometry approach to identify the RNF128 interacting proteins.**

**Supplementary Fig. 4 RNF128 regulates the expression of inflammatory cytokines and MPO in BALF neutrophils.** **A-E** The levels of CD11b, MPO, TNF-α, IL-1ß, and IL-6 mRNAs in neutrophils from BALF were detected using Q-PCR. The statistical significance was evaluated using one-way ANOVA with Newman–Keuls post-hoc test or Student’s t-test. *P < 0.05; **P < 0.01; ***P < 0.001.

**Supplementary Fig. 5 RNF128 gene expressions in the lung tissue.** The mRNA expression of RNF128 in the lung of AAV9-Vector and AAV9-RNF128 mice after LPS treatment. The data are presented as mean values ± SD. ^***^*P*<0.001, Student’s *t-*test

**Table 1. The RNF128-involved pathway was identified using IP-Mass**

Immunoprecipitated proteins were identified using liquid chromatography-tandem mass spectroscopy (LC-MS/MS). Forty-eight unique proteins were at least 2-fold more abundant than in the WT control. Sixteen of these proteins are listed in the table.

| **Accession** | Description |  | **WT-LPS** | **WT** | **Fold Change** |
| --- | --- | --- | --- | --- | --- |
| **P11247** | **Myeloperoxidase OS=Mus musculus OX=10090 GN=Mpo PE=1 SV=2 - [PERM_MOUSE]** | **Mpo** | **2656.150413** | **102.1480795** | **26.00294031** |
| **Q9JIK5** | Nucleolar RNA helicase 2 OS=Mus musculus OX=10090 GN=Ddx21 PE=1 SV=3 - [DDX21_MOUSE] | Ddx21 | 566.2804627 | 28.04 | 20.1954516 |
| **P20918** | Plasminogen OS=Mus musculus OX=10090 GN=Plg PE=1 SV=3 - [PLMN_MOUSE] | Plg | 560.4548281 | 29.03 | 19.30605677 |
| **Q9ET01** | Glycogen phosphorylase, liver form OS=Mus musculus OX=10090 GN=Pygl PE=1 SV=4 - [PYGL_MOUSE] | Pygl | 675.6869767 | 69.15111269 | 9.771165645 |
| **P61979** | Heterogeneous nuclear ribonucleoprotein K OS=Mus musculus OX=10090 GN=Hnrnpk PE=1 SV=1 - [HNRPK_MOUSE] | Hnrnpk | 513.0475359 | 59.73333333 | 8.588965445 |
| **Q9WUU8** | TNFAIP3-interacting protein 1 OS=Mus musculus OX=10090 GN=Tnip1 PE=1 SV=1 - [TNIP1_MOUSE] | Tnip1 | 357.349945 | 45.72789999 | 7.814702733 |
| **Q99PL5** | Ribosome-binding protein 1 OS=Mus musculus OX=10090 GN=Rrbp1 PE=1 SV=2 - [RRBP1_MOUSE] | Rrbp1 | 458.6563932 | 61.00301438 | 7.518585727 |
| **Q9CZX8** | 40S ribosomal protein S19 OS=Mus musculus OX=10090 GN=Rps19 PE=1 SV=3 - [RS19_MOUSE] | Rps19 | 287.489367 | 38.87 | 7.396176151 |
| **Q99K48** | Non-POU domain-containing octamer-binding protein OS=Mus musculus OX=10090 GN=Nono PE=1 SV=3 - [NONO_MOUSE] | Nono | 311.08064 | 43.94 | 7.079668638 |
| **P10126** | Elongation factor 1-alpha 1 OS=Mus musculus OX=10090 GN=Eef1a1 PE=1 SV=3 - [EF1A1_MOUSE] | Eef1a1 | 473.3300346 | 97.66 | 4.846713441 |
| **P26041** | Moesin OS=Mus musculus OX=10090 GN=Msn PE=1 SV=3 - [MOES_MOUSE] | Msn | 1264.85134 | 275.8477857 | 4.58532352 |
| **P26039** | Talin-1 OS=Mus musculus OX=10090 GN=Tln1 PE=1 SV=2 - [TLN1_MOUSE] | Tln1 | 199.3702293 | 46.18 | 4.317241864 |
| **O08692** | Neutrophilic granule protein OS=Mus musculus OX=10090 GN=Ngp PE=1 SV=1 - [NGP_MOUSE] | Ngp | 1339.731731 | 324.8263491 | 4.124455219 |
| **Q8VDD5** | Myosin-9 OS=Mus musculus OX=10090 GN=Myh9 PE=1 SV=4 - [MYH9_MOUSE] | Myh9 | 763.8241756 | 195.3123617 | 3.910782548 |
| **P62245** | 40S ribosomal protein S15a OS=Mus musculus OX=10090 GN=Rps15a PE=1 SV=2 - [RS15A_MOUSE] | Rps15a | 171.2987103 | 45.35625824 | 3.776738138 |
| **Q8K2I3** | Dimethylaniline monooxygenase [N-oxide-forming] 2 OS=Mus musculus OX=10090 GN=Fmo2 PE=1 SV=3 - [FMO2_MOUSE] | Fmo2 | 111.9302569 | 31.86906198 | 3.51219176 |
| **P20029** | Endoplasmic reticulum chaperone BiP OS=Mus musculus OX=10090 GN=Hspa5 PE=1 SV=3 - [BIP_MOUSE] | Hspa5 | 408.7148581 | 121.53 | 3.363077907 |
| **Q6PDM2** | Serine/arginine-rich splicing factor 1 OS=Mus musculus OX=10090 GN=Srsf1 PE=1 SV=3 - [SRSF1_MOUSE] | Srsf1 | 368.0803336 | 113.558529 | 3.241327065 |
| **P62918** | 60S ribosomal protein L8 OS=Mus musculus OX=10090 GN=Rpl8 PE=1 SV=2 - [RL8_MOUSE] | Rpl8 | 402.9989846 | 127.3756428 | 3.163862224 |
| **Q8R081** | Heterogeneous nuclear ribonucleoprotein L OS=Mus musculus OX=10090 GN=Hnrnpl PE=1 SV=2 - [HNRPL_MOUSE] | Hnrnpl | 514.2045533 | 164.5514161 | 3.124886832 |
| **P01872** | Immunoglobulin heavy constant mu OS=Mus musculus OX=10090 GN=Ighm PE=1 SV=2 - [IGHM_MOUSE] | Ighm | 478.7699937 | 158.91 | 3.012837415 |
| **Q02105** | Complement C1q subcomponent subunit C OS=Mus musculus OX=10090 GN=C1qc PE=1 SV=2 - [C1QC_MOUSE] | C1qc | 89.02385808 | 31.2 | 2.853328785 |
| **P62849** | 40S ribosomal protein S24 OS=Mus musculus OX=10090 GN=Rps24 PE=1 SV=1 - [RS24_MOUSE] | Rps24 | 455.8471599 | 162.5019545 | 2.805179553 |
| **P01864** | Ig gamma-2A chain C region secreted form OS=Mus musculus OX=10090 PE=1 SV=1 - [GCAB_MOUSE] |  | 468.2990245 | 167.4 | 2.797485212 |
| **Q8BMF4** | Dihydrolipoyllysine-residue acetyltransferase component of pyruvate dehydrogenase complex, mitochondrial OS=Mus musculus OX=10090 GN=Dlat PE=1 SV=2 - [ODP2_MOUSE] | Dlat | 509.7281186 | 186.46 | 2.73371296 |
| **Q9D0T1** | NHP2-like protein 1 OS=Mus musculus OX=10090 GN=Snu13 PE=1 SV=4 - [NH2L1_MOUSE] | Snu13 | 179.43 | 66.69 | 2.690508322 |
| **O70133** | ATP-dependent RNA helicase A OS=Mus musculus OX=10090 GN=Dhx9 PE=1 SV=2 - [DHX9_MOUSE] | Dhx9 | 292.4390632 | 111.8318968 | 2.614987955 |
| **P63276** | 40S ribosomal protein S17 OS=Mus musculus OX=10090 GN=Rps17 PE=1 SV=2 - [RS17_MOUSE] | Rps17 | 156.7504083 | 61.14 | 2.563794706 |
| **P27546** | Microtubule-associated protein 4 OS=Mus musculus OX=10090 GN=Map4 PE=1 SV=3 - [MAP4_MOUSE] | Map4 | 115.58 | 45.36 | 2.548059965 |
| **Q9JJI8** | 60S ribosomal protein L38 OS=Mus musculus OX=10090 GN=Rpl38 PE=1 SV=3 - [RL38_MOUSE] | Rpl38 | 107.4458462 | 42.71 | 2.515707006 |
| **P01899** | H-2 class I histocompatibility antigen, D-B alpha chain OS=Mus musculus OX=10090 GN=H2-D1 PE=1 SV=2 - [HA11_MOUSE] | H2-D1 | 185.7113999 | 74.82 | 2.482109061 |
| **Q8VIJ6** | Splicing factor, proline- and glutamine-rich OS=Mus musculus OX=10090 GN=Sfpq PE=1 SV=1 - [SFPQ_MOUSE] | Sfpq | 531.8918203 | 220.4183318 | 2.413101559 |
| **Q8VEK3** | Heterogeneous nuclear ribonucleoprotein U OS=Mus musculus OX=10090 GN=Hnrnpu PE=1 SV=1 - [HNRPU_MOUSE] | Hnrnpu | 1144.404106 | 478.7680874 | 2.390309915 |
| **Q9R0P5** | Destrin OS=Mus musculus OX=10090 GN=Dstn PE=1 SV=3 - [DEST_MOUSE] | Dstn | 94.6 | 39.66333333 | 2.385074376 |
| **P50404** | Pulmonary surfactant-associated protein D OS=Mus musculus OX=10090 GN=Sftpd PE=1 SV=1 - [SFTPD_MOUSE] | Sftpd | 249.7905069 | 109.321253 | 2.284921734 |
| **Q8BTM8** | Filamin-A OS=Mus musculus OX=10090 GN=Flna PE=1 SV=5 - [FLNA_MOUSE] | Flna | 844.3285904 | 372.4891434 | 2.266719998 |
| **P62281** | 40S ribosomal protein S11 OS=Mus musculus OX=10090 GN=Rps11 PE=1 SV=3 - [RS11_MOUSE] | Rps11 | 323.7022669 | 143.4275606 | 2.256904222 |
| **P61327** | Protein mago nashi homolog OS=Mus musculus OX=10090 GN=Magoh PE=2 SV=1 - [MGN_MOUSE] | Magoh | 111.886991 | 49.57651523 | 2.256854692 |
| **P62852** | 40S ribosomal protein S25 OS=Mus musculus OX=10090 GN=Rps25 PE=1 SV=1 - [RS25_MOUSE] | Rps25 | 258.4070323 | 116.2386753 | 2.223072756 |
| **P48962** | ADP/ATP translocase 1 OS=Mus musculus OX=10090 GN=Slc25a4 PE=1 SV=4 - [ADT1_MOUSE] | Slc25a4 | 198.1835486 | 89.31673657 | 2.218884794 |
| **P43276** | Histone H1.5 OS=Mus musculus OX=10090 GN=Hist1h1b PE=1 SV=2 - [H15_MOUSE] | Hist1h1b | 486.4603075 | 223.1484272 | 2.179985373 |
| **P01867** | Ig gamma-2B chain C region OS=Mus musculus OX=10090 GN=Igh-3 PE=1 SV=3 - [IGG2B_MOUSE] | Igh-3 | 883.5515727 | 408.4762325 | 2.163042797 |
| **P63017** | Heat shock cognate 71 kDa protein OS=Mus musculus OX=10090 GN=Hspa8 PE=1 SV=1 - [HSP7C_MOUSE] | Hspa8 | 639.4902806 | 295.9818624 | 2.160572528 |
| **Q6ZWV3** | 60S ribosomal protein L10 OS=Mus musculus OX=10090 GN=Rpl10 PE=1 SV=3 - [RL10_MOUSE] | Rpl10 | 604.5828163 | 285.6226998 | 2.116718373 |
| **P01837** | Immunoglobulin kappa constant OS=Mus musculus OX=10090 GN=Igkc PE=1 SV=2 - [IGKC_MOUSE] | Igkc | 611.1369241 | 295.1554889 | 2.070559237 |
| **P51410** | 60S ribosomal protein L9 OS=Mus musculus OX=10090 GN=Rpl9 PE=2 SV=2 - [RL9_MOUSE] | Rpl9 | 165.2937513 | 80.13333333 | 2.062734001 |
| **Q6ZWY3** | 40S ribosomal protein S27-like OS=Mus musculus OX=10090 GN=Rps27l PE=1 SV=3 - [RS27L_MOUSE] | Rps27l | 251.7 | 124.87 | 2.015696324 |
| **Q9JHL1** | Na(+)/H(+) exchange regulatory cofactor NHE-RF2 OS=Mus musculus OX=10090 GN=Slc9a3r2 PE=1 SV=2 - [NHRF2_MOUSE] | Slc9a3r2 | 58.31333333 | 28.93 | 2.015670008 |

| **Supplementary Table 2 \| Primers used for Q-PCR analysis** | | |
| --- | --- | --- |
| **Primers** | **Forward sequence** | **Reverse sequence** |
| **RNF128** | *5’-GCGTCTGGAGCCGTCATCTTTA-3’* | *5’-GGGCCATGTTTTTTCCCTACTTCTAT-3’* |
| **GAPDH** | *5’-TTCACCACCATGGAGAAGGC-3’* | *5’-GATGGCATGGACTGTGGTC-3’* |
| **TNF-α** | *5’-TAGCCCACGTCGTAGCAAAC-3’* | *5’- GATAGCAAATCGGCTGACGG-3’* |
| **IL-1β** | *5’- CCCAAAAGATGAAGGGCTGC-3’* | *5’- AAGGTCCACGGGAAAGACAC-3’* |
| **MPO** | *5’- TCCCACTCAGCAAGGTCTT-3’* | *5’- TAAGAGCAGGCAAATCAAG-3’* |
| **IL-6** | *5’- TCTAATTCATATCTTCAACCAAGA-3’* | *5’- TGGTCCTTAGCCACTCCTTC-3’* |
