# Supplementary figures and images for: RNF128 regulates neutrophil infiltration and myeloperoxidase functions to prevent acute lung injury
Source: Cell Death Dis. 2023 Jun 21;14(6):369. doi: 10.1038/s41419-023-05890-1 (PMC10284794; doi:10.1038/s41419-023-05890-1)

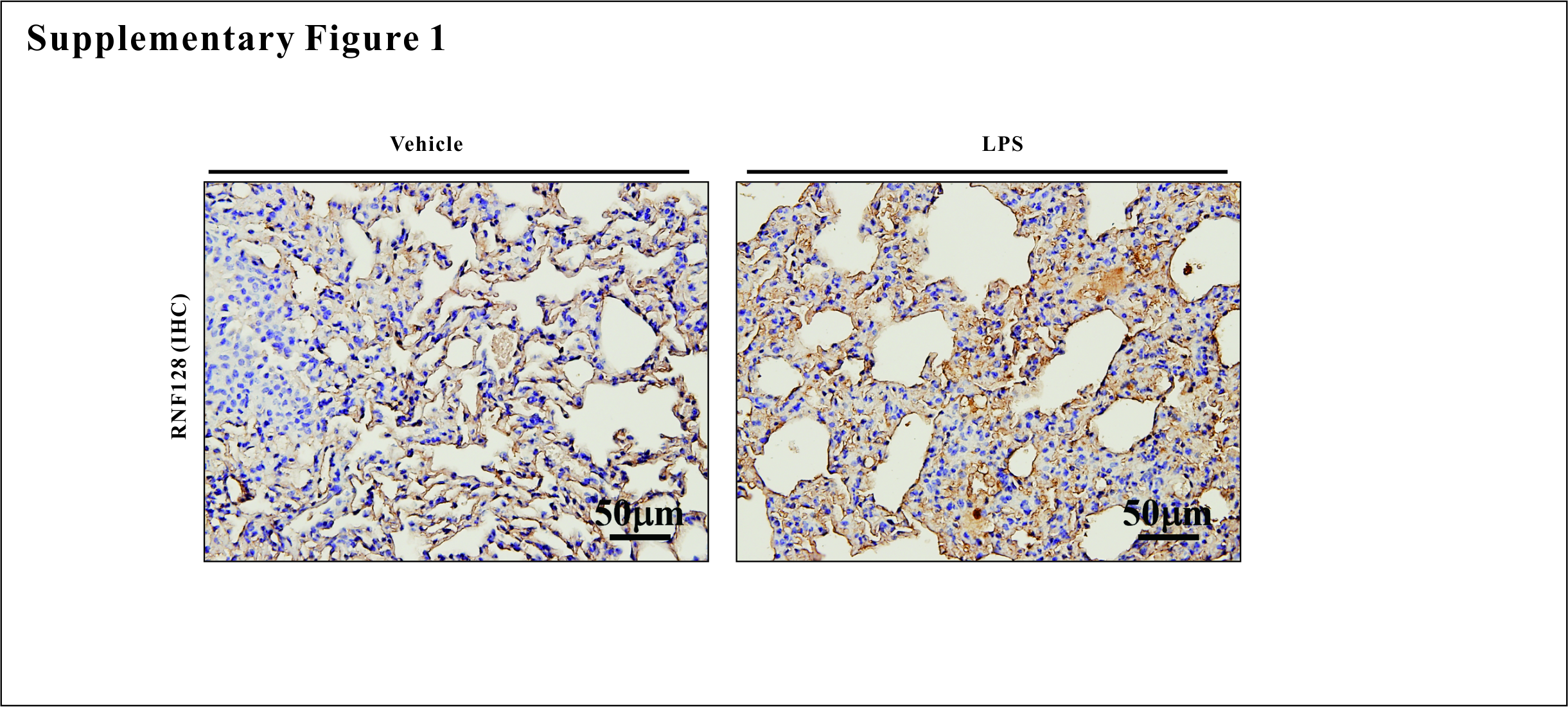

Supplement: Supplementary file 3 — Supplementary Figure 1 [file 41419_2023_5890_MOESM3_ESM.tif]

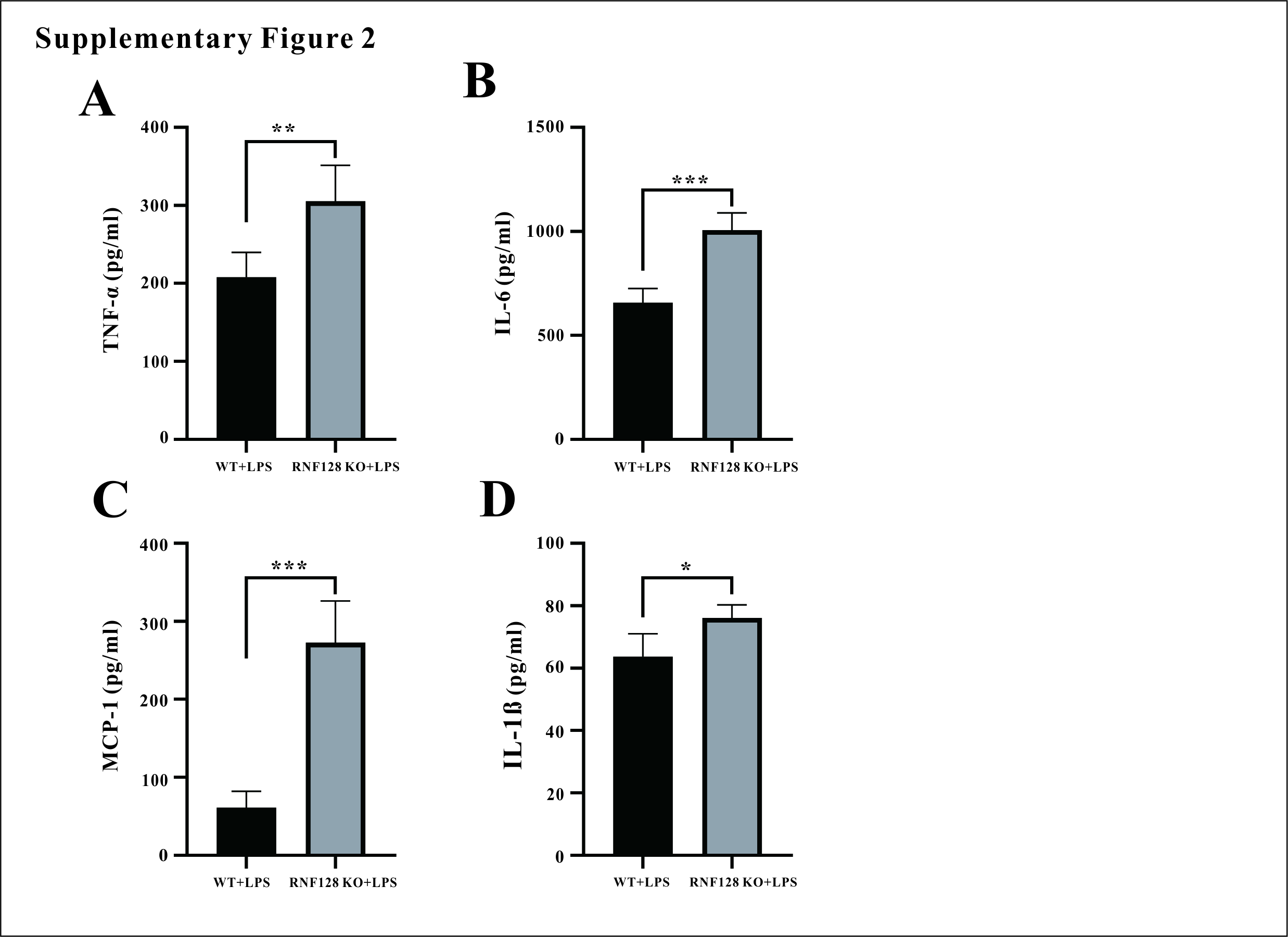

Supplement: Supplementary file 4 — Supplementary Figure 2 [file 41419_2023_5890_MOESM4_ESM.tif]

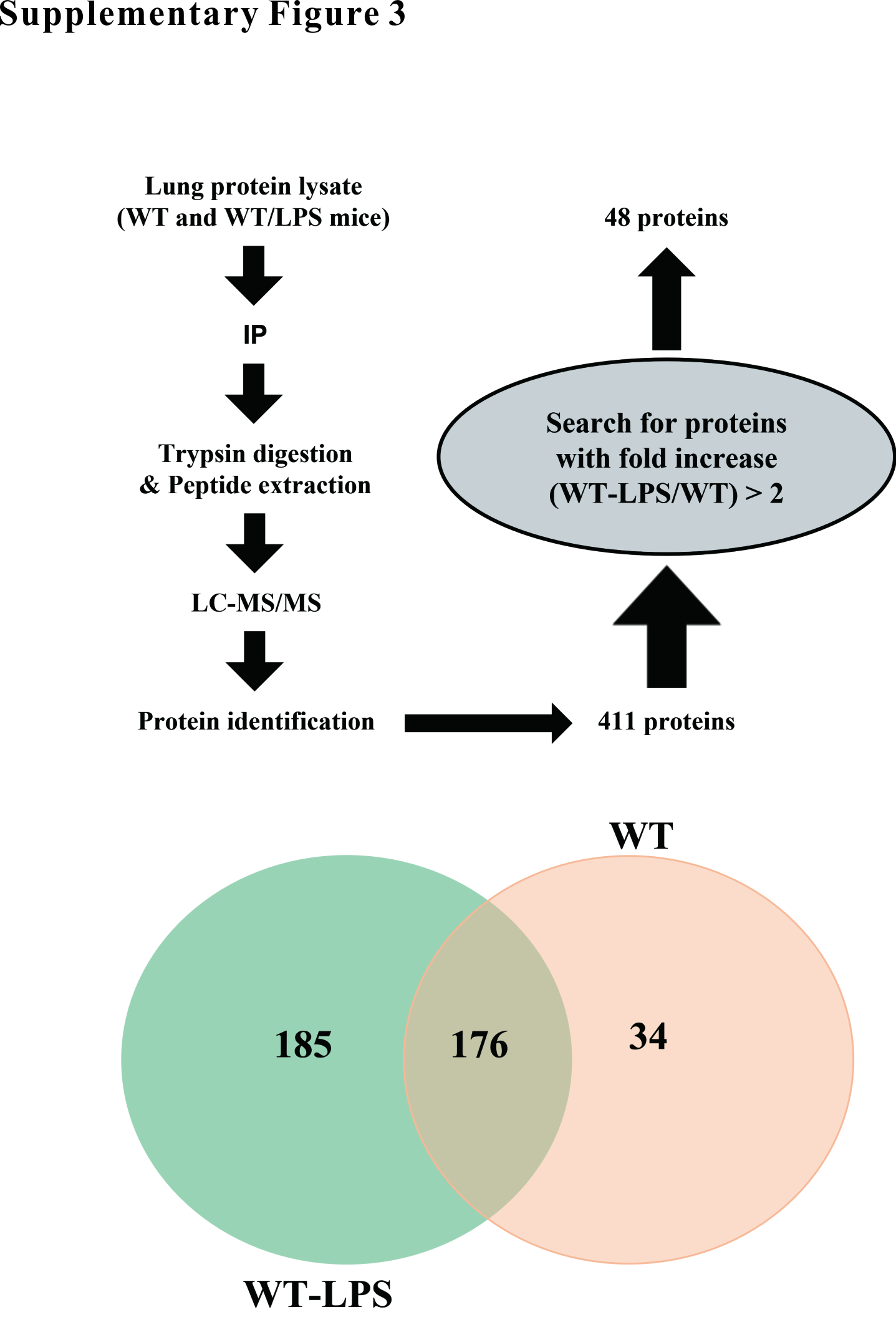

Supplement: Supplementary file 5 — Supplementary Figure 3 [file 41419_2023_5890_MOESM5_ESM.tif]

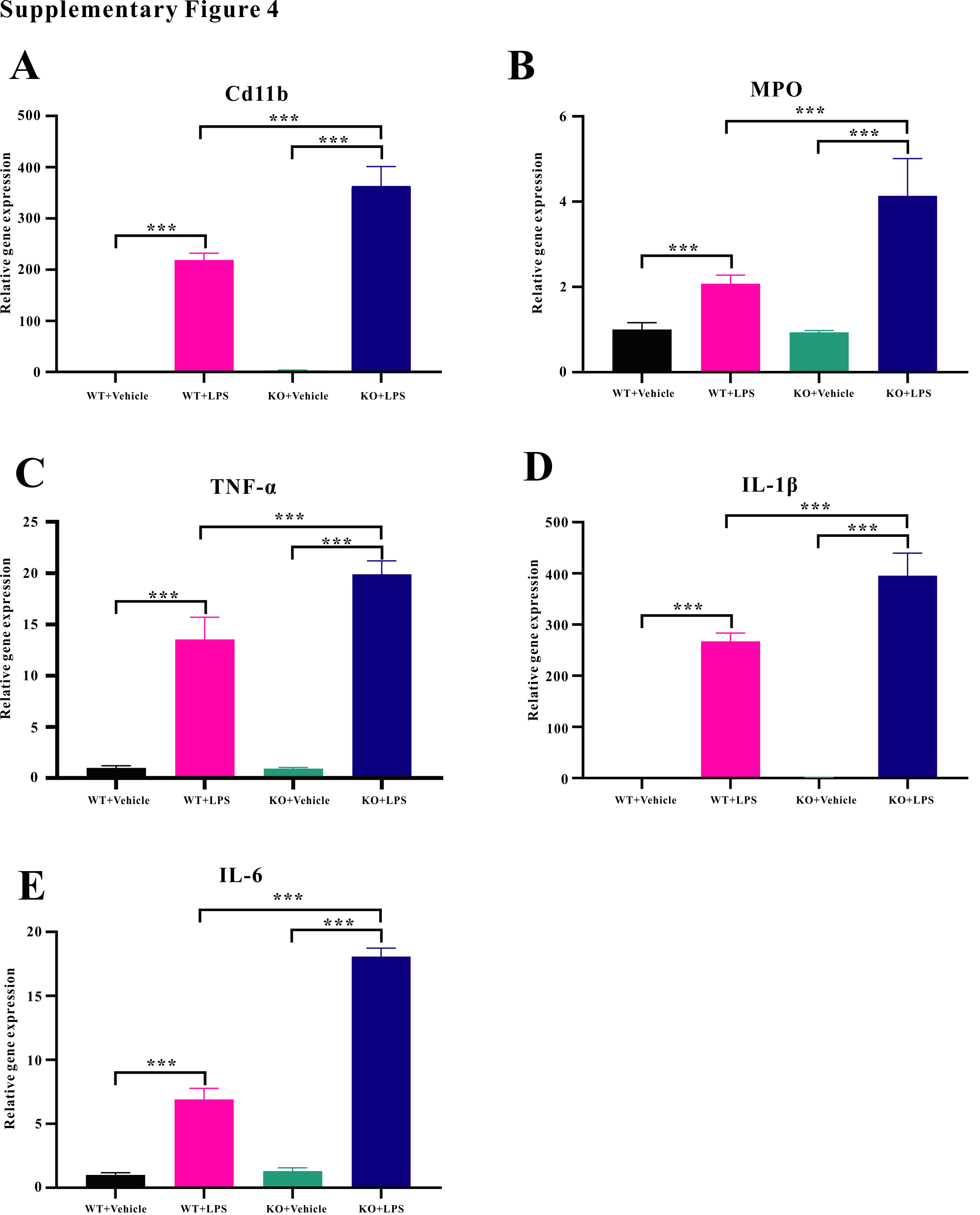

Supplement: Supplementary file 6 — Supplementary Figure 4 [file 41419_2023_5890_MOESM6_ESM.tif]

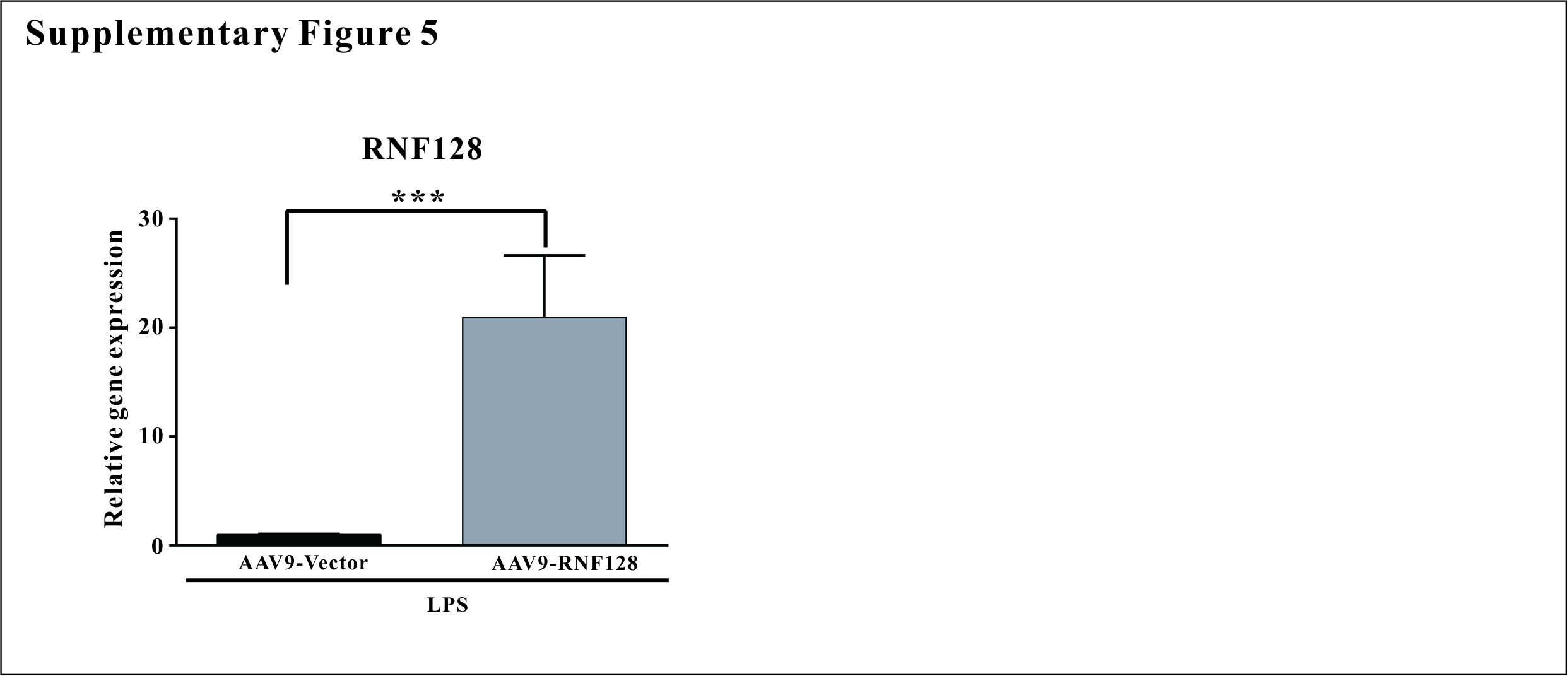

Supplement: Supplementary file 7 — Supplementary Figure 5 [file 41419_2023_5890_MOESM7_ESM.tif]
